# Predicting the stability of large structured food webs

**DOI:** 10.1038/ncomms8842

**Published:** 2015-07-22

**Authors:** Stefano Allesina, Jacopo Grilli, György Barabás, Si Tang, Johnatan Aljadeff, Amos Maritan

**Affiliations:** 1 Department of Ecology & Evolution, University of Chicago, 1101 E. 57th st., Chicago, IL 60637, USA; 2 Computation Institute, University of Chicago, Chicago, IL 60637, USA; 3 Department of Physics and Astronomy ‘Galileo Galilei', Università degli Studi di Padova, via Marzolo 8, I-35131 Padova, Italy; 4 Department of Statistics, University of Chicago, 5734S. University Avenue, Chicago, IL 60637, USA; 5 Department of Neurobiology, University of Chicago, 947 E. 58th st., Chicago, IL 60637, USA

## Abstract

The stability of ecological systems has been a long-standing focus of ecology. Recently, tools from random matrix theory have identified the main drivers of stability in ecological communities whose network structure is random. However, empirical food webs differ greatly from random graphs. For example, their degree distribution is broader, they contain few trophic cycles, and they are almost interval. Here we derive an approximation for the stability of food webs whose structure is generated by the cascade model, in which ‘larger' species consume ‘smaller' ones. We predict the stability of these food webs with great accuracy, and our approximation also works well for food webs whose structure is determined empirically or by the niche model. We find that intervality and broad degree distributions tend to stabilize food webs, and that average interaction strength has little influence on stability, compared with the effect of variance and correlation.

## Abstract (web summary)

Understanding the dynamics of empirical food webs is of central importance for predicting the stability of ecological communities. Here Allesina *et al.* derive an approximation to accurately predict the stability of large food webs whose structure is built using the cascade model.

## Graphical abstract

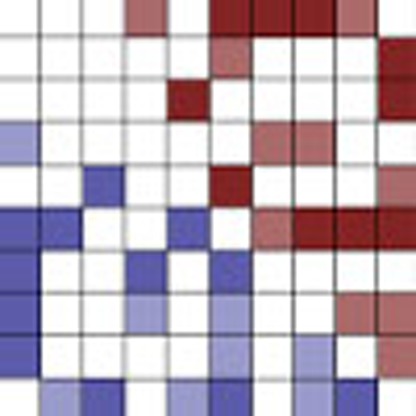

The stability of large ecological systems has been investigated for more than 40 years^1^. The interest in this subject was sparked by a short article by Robert May^1^, who was able to show that large ecosystems with random interactions would invariably be unstable, with potential consequences for biodiversity maintenance. To obtain this result, May employed basic tools of random matrix theory, and recent advances in this area^2^,^3^ allowed for an extension of May's result to more general cases^4^,^5^—effectively identifying the main drivers of stability in ecological communities.

All these findings hinge on an important assumption that the network structure describing who interacts with whom in an ecosystem is random^4^,^5^, that is, any two species have the same probability of interacting, irrespective of species identity. However, the empirical food webs collected thus far display major departures from the structure of random graphs^6^. For example, in empirical webs the degree distribution, describing the number of partners each species interact with, is much broader^6^ than in random graphs; the webs contain only a handful of trophic cycles^7^ (in which, for example, species *a* consumes *b*, *b* consumes *c* and *c* consumes *a*), while random graphs with the same number of links would contain many more; finally, empirical webs are almost interval—there is a way to order all species such that consumers tend to prey on consecutive species in the hierarchy^8^.

To overcome this limitation, we derive an approximation for the stability of food webs whose structure is generated by the cascade model^9^, which assumes that species can be ordered such that ‘larger' species consume ‘smaller' ones. We sample the strength of interaction between consumers and resources from an empirical distribution, obtained via body-size scaling theory^5^. We show numerically that our approximation estimates the stability of these food webs with great accuracy, and that similar results are obtained when we generate food webs starting from empirical data, or when using the niche model^10^. We show that intervality and broad degree distributions tend to stabilize food webs, and we highlight a counterintuitive result: although research on the relationship between stability and the distribution of interaction strengths has historically focused on average strength^11^,^12^,^13^,^14^, we show that its role in determining stability is small, compared with that of variance and correlation.

## Results

### Constructing the community matrix

We want to determine the real part of the leading (‘rightmost') eigenvalue of the community matrix **M**, Re(*λ*_**M**,1_), which is the key for the local asymptotic stability of the ecological system. In fact, the community matrix^1^,^15^ determines the effects of one species on another around a feasible equilibrium point: if all the eigenvalues of **M** have a negative real part, the equilibrium is locally stable—small perturbations will be buffered (Supplementary Note 1). The study of community matrices has a long history in ecology, but so far methods relying on large random matrices^4^,^5^ have not been able to account for realistic food web structure, and were based on the simplifying assumption of a completely random network, in which every species has the same probability of consuming any other.

Here we study the matrix **M** constructed in the following way. First, an *S* × *S* adjacency matrix **K** is built according to the cascade model^9^: the species are ordered from 1 to *S*, and each species *j* has probability *C* of consuming each of the preceding species. A coefficient *K*_*ij*_=1 means that species *i* is consumed by *j*. Then, we build the community matrix **M** by independently sampling each pair of coefficients (*M*_*ij*_,*M*_*ji*_)_*i*<*j*_ from the bivariate distribution *Z*=(*X*,*Y*) whenever *K*_*ij*_=1. For simplicity, we leave *M*_*ii*_=0. Setting all diagonal coefficients to −*d* would simply shift all the eigenvalues (*λ*_*i*′_=*λ*_*i*_−*d*), and sampling the diagonal coefficients from a distribution with mean −*d* and a given variance would yield qualitatively the same results, provided that the variance is not large compared with that of the off-diagonal coefficients^5^. In this setting, we can think of Re(*λ*_**M**,1_) as the minimum amount of self-regulation we would have to impose on each species to make the system stable^11^.

Because the pairs (*M*_*ij*_,*M*_*ji*_) model the effect of the consumer on the resource (*M*_*ij*_) and that of the resource on the consumer (*M*_*ji*_), we have *M*_*ij*_<0 and *M*_*ji*_>0 whenever *K*_*ij*_=1. Thus, we assume *Z*=(*X*,*Y*) to be a bivariate distribution with marginal means *μ*_x_ and *μ*_y_, Var(*X*)=*σ*_x_^2^, Var(*Y*)=*σ*_y_^2^, and Cov(*X*, *Y*)=*ρ*_xy_*σ*_x_*σ*_y_, where *μ*_x_<0, and *μ*_y_>0 (see Methods section).

The matrix **M** then contains non-positive coefficients in the upper-triangular part (either 0 when *K*_*ij*_=0, or negative when *K*_*ij*_=1). Similarly, the lower-triangular part of the matrix contains only non-negative coefficients. We denote by *μ*_U_ and *μ*_L_ the means of the upper- and lower-triangular coefficients; by *σ*_U_^2^ and *σ*_L_^2^ the variances; and by *ρ*_UL_*σ*_U_*σ*_L_ the covariance.

### Derivation strategy

**Figure 1:**
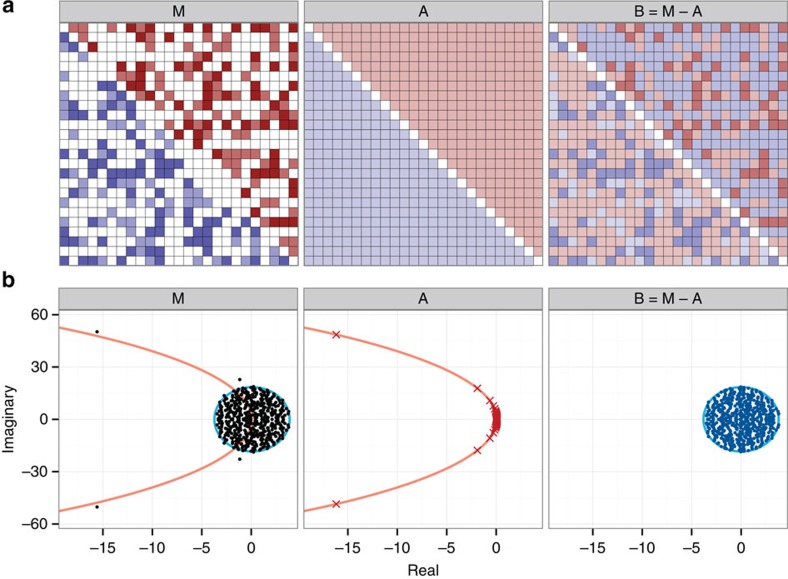
The derivation strategy. (**a**) The matrix **M** has non-positive coefficients in the upper-triangular part, and non-negative coefficients in the lower-triangular part, as we would expect for community matrices of food webs built according to the cascade model (colours denote the sign and magnitude of the coefficients, with shades of blue for positive coefficients and shades of red for negative ones). **M**=**A**+**B**, where **A** has upper-triangular coefficients equal to the mean of the upper-triangular part of **M**, and lower-triangular coefficients equal to the mean of the lower-triangular part of **M** and **B**=**M**−**A**. (**b**) The spectrum of the three matrices with *S*=500, *C*=0.25. The eigenvalues of **A** (red crosses) fall on a the curve defining a circle (orange), while those of **B** (dark blue) are approximately uniform over an ellipse (light blue) centred at (0,0). The eigenvalues of **M** either fall close to the curve obtained for **A**, or in the ellipse found for **B**, centred at (Re(*λ*_**A**,1_)≈0.08,0), rather than (0,0).

Having shown how **M** is built, we now illustrate the strategy we use to find the distribution of its eigenvalues. First, we decompose the matrix into the sum of two matrices, **M**=**A**+**B**, where **A** is a deterministic matrix whose upper-triangular coefficients are all equal to *μ*_U_, and all the lower-triangular to *μ*_L_. **B** is obtained by difference, **B**=**M**−**A**, and therefore its coefficients are described by a bivariate distribution with means 0 and covariance matrix identical to that found for the coefficients of **M**. Matrix **A** models the ‘signal', and **B** the ‘noise' (Fig. 1). We then studied the spectrum of **A** and **B** separately.

For **A**, one can show (see Methods section) that all the eigenvalues fall on the curve describing a circle in the complex plane with center (*c*_*A*_,0) and radius *r*_*A*_. When −*μ*_U_>*μ*_L_, that is, negative effects are stronger than positive ones, the bulk of the eigenvalues of **A** have positive real part, and a few eigenvalues with large modulus have negative real part (Fig. 1). In this case, Re(*λ*_**A**,1_)≈*r*_*A*_+*c*_*A*_, and 0≤Re(*λ*_**A**,1_)≤−*μ*_U_ for any choice of size and parameters (Supplementary Note 3).

For *σ*_L_^2^=*σ*_U_^2^=*σ*^2^, the eigenvalues of **B** would follow the elliptic law^3^, and thus, for large *S*, they would be approximately uniformly distributed over an ellipse in the complex plane centred at (0,0), with horizontal semi-axis approximately (*Sσ*^2^)^1/2^(1+*ρ*) and vertical semi-axis approximately (*Sσ*^2^)^1/2^(1−*ρ*). Here we conjecture that even in the more general case of *σ*_L_^2^≠*σ*_U_^2^, the eigenvalues of **B** are approximately uniform in an ellipse centred at (0,0) with horizontal semi-axis *r*_*h*,*B*_, and vertical semi-axis *r*_*v*,*B*_ (see Methods section).

Having shown that when −*μ*_U_>*μ*_L_, Re(*λ***A**_,1_)≈*r*_*A*_+*c*_*A*_, and Re(*λ*_**B**,1_)≈*r*_*h*,*B*_, we take the last approximation: Re(*λ*_**M**,1_)≈Re(*λ*_**A**,1_)+Re(*λ*_**B**,1_). In fact, the eigenvalues of **M** fall either close to the curve found for **A**, or in the ellipse found for **B**, centred at (Re(*λ*_**A**,1_),0) instead of (0,0) (Fig. 1). This type of approximation is known to be accurate for symmetric matrices (following Weyl's inequality^16^), but our results suggest that it is well suited for the matrices studied here as well, provided that the variances *σ*_L_^2^ and *σ*_U_^2^ are sufficiently large compared with *μ*_L_ and *μ*_U_ (Supplementary Note 3).

### Numerical results

**Figure 2:**
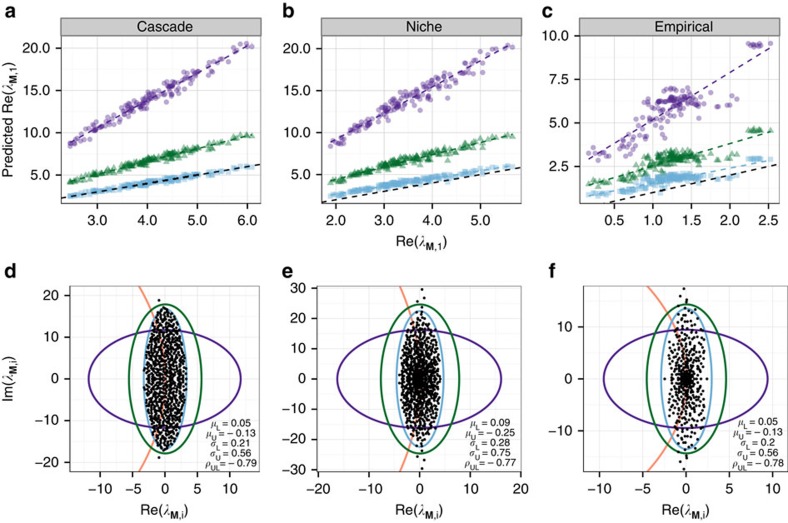
Accuracy of the approximation. (**a**) We parameterized 150 community matrices with structure determined by the cascade model. The predicted Re(*λ*_**M**,1_) (real part of the leading eigenvalue of **M**, light blue squares) is much closer to the observed value than when approximating it using the method by Tang *et al.*^5^ (green triangles) or using May's criterion^1^ (purple dots). The coloured dashed lines are the best-fitting linear models, and the black dashed line is the bisector of the first quadrant, marking perfect predictions. (**b**) The bulk of the eigenvalues of **M** for one of the matrices. The light blue ellipse is that found for **B**, but centred at (Re(*λ*_**A**,1_),0) (the red line indicates the curve describing the circle where we expect the eigenvalues of **A** to fall). The green ellipse is that predicted according to Tang *et al.*^5^, and the purple circle is that predicted using May's criterion^1^. (**b** and **e**) The same plots for matrices built using the niche model. (**c** and **f**) The same plots but for matrices built parameterizing 10 times each of 15 empirical food webs.

To test the quality of our approximation, we built 150 adjacency matrices **K** using the cascade model^9^. The size of the matrix was randomly chosen among {500, 750, 1,000}, and the probability of interaction *C* was sampled uniformly between 0.1 and 0.3. We sampled the pairs (*M*_*ij*_,*M*_*ji*_) independently from the empirical distribution *Z* whenever *K*_*ij*_=1. The results are presented in Fig. 2. The approximation is very accurate, and clearly superior to what expected following the derivations by May^1^ or Tang *et al.*^5^.

We then built 150 adjacency matrices using the niche model^10^, which can generate trophic cycles, so that there is no way to order the species such that all the coefficients in the upper-triangular part of **M** are non-positive. Hence, even knowing the distribution *Z*, we need to find a way to calculate *μ*_U_, *μ*_L_ and so on. Clearly, the eigenvalues of **M** do not change when we sort the species in different ways, but, because our approximation makes explicit use of the coefficients in the upper- and lower-triangular parts of **M**, each ordering of the species would yield a different approximation. To choose the ‘best' approximation, we sorted the species in the adjacency matrix **K** so that the minimum number of non-zero coefficients were present in the lower-triangular part of the matrix, and then used this order to parameterize the approximation (Supplementary Note 2). Results show that the approximation is also excellent for the niche model, even though the matrices built in this way are slightly more likely to be stable than those built using the cascade model (Fig. 2).

Finally, we took 15 large empirical food webs (Supplementary Note 2) and parameterized each food web 10 times. Also in this case, we sorted the adjacency matrix **K** to obtain the ‘most upper-triangular' configuration prior to calculating the approximation. Despite the fact that empirical networks are quite different from those generated by the cascade model (for example, containing ‘modules'^17^ and having broader degree distributions^6^), the approximation is clearly superior to previous approaches, even though it still tends to over-estimate the actual Re(*λ*_**M**,1_).

### Effect of network properties

Having an analytic expectation for Re(*λ*_**M**,1_) allows us to investigate which particular features of network structure are stabilizing. For example, the networks produced by the niche model differ from those generated by the cascade model in three main aspects. First, although trophic cycles are forbidden in the cascade model, the niche model typically produces networks with a handful of trophic cycles. Second, in a food web produced by the niche model, we can always order the species such that each predator consumes prey that are adjacent in the hierarchy (for example, this would be the case if each predator were to prey upon all the species falling in a certain interval of sizes^18^), a property known as ‘intervality'. Intervality is rarely attained by the cascade model, especially for large webs^9^. Finally, the degree distributions (that is, number of predators per prey, and number of prey per predator) differ substantially between the networks produced by the two models (starting from the same parameters).

**Figure 3:**
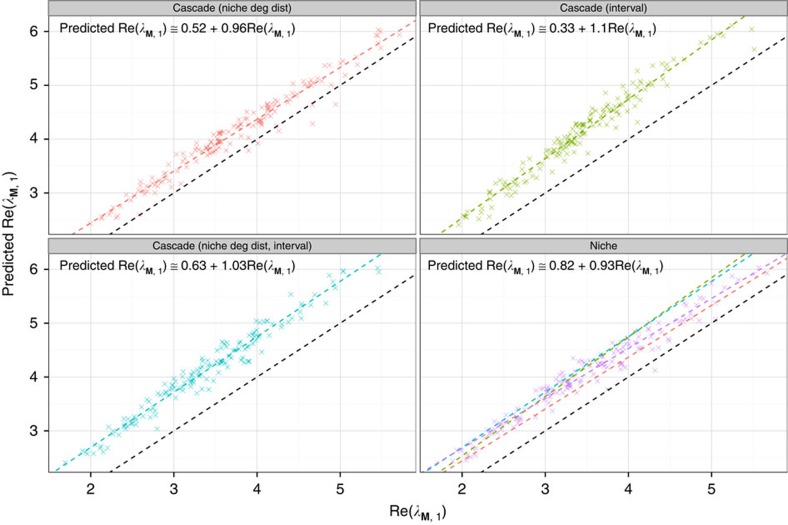
Cascade model variants. Simulations performed as for Fig. 2, but with variants of the cascade model. Given that our prediction matches Re(*λ*_**M**,1_) (the real part of the leading eigenvalue of **M**) very closely for matrices built using the cascade model, any deviation displayed here necessarily descends from the different structural models employed. Points above the dashed black line (the identity line) indicate networks with smaller Re(*λ*_**M**,1_) than that we would predict using the cascade model approximation, indicating a stabilizing effect of the structure with respect to the cascade model. For each model, we display the predicted versus observed Re(*λ*_**M**,1_), as well as the best-fitting linear model. All lines are reported in the Niche model panel for ease of comparison. In all the cases, the effect of the variation is stabilizing, with intervality having a stronger effect than the degree distribution.

To test whether these features can account for the small discrepancy between our expectation and that found in simulations, we built three variants of the cascade model: (i) a version of the cascade model producing the same degree distribution for the consumers as that of the niche model; (ii) a version producing interval food webs; (iii) a version yielding the same consumer degree distribution as the niche model, and producing interval food webs (that is, a cycle-less niche model). In Fig. 3 we show that all these modifications are slightly stabilizing, making the matrices built using these variants as likely to be stable as those for the niche model. Similarly, modifying the cascade model so that it matches the degree distribution of a given empirical network recapitulates the small deviation we observe between the predicted and observed Re(*λ*_**M**,1_) for matrices generated using empirical food web structures (Supplementary Note 4).

### The case of strong positive effects

So far, we have concentrated on the case of −*μ*_U_>*μ*_L_ meaning that negative interactions are on an average stronger than the positive ones. This is what is typically found in food webs—due to the low efficiency of transformation of prey into predators. When this is not the case, the eigenvalue distribution of **M** is ‘flipped' around the imaginary axis (that is, the distribution is like that in the bottom-left panel of Fig. 1, but with the x-axis reversed), such that a pair of large complex roots determines the stability of the system (Supplementary Note 3). This observation is sufficient to make a suggestive prediction: large systems in which the positive effects dominate the negative ones will likely lose stability through a Hopf bifurcation (two complex roots crossing the imaginary axis), typically giving rise to limit cycles. For a simple Lotka–Volterra model, a pair of coefficients of matrix **M** modelling a resource–consumer interaction can be written as *M*_*ij*_=−*β*_*ij*_*N*_i_* and 
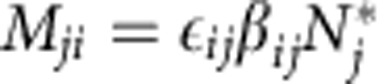
, where *β*_*ij*_ is the attack rate of *j* on *i*, *N*_i_* is the equilibrium biomass of resource *i*, and 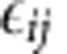 is the conversion efficiency of resources into consumers. In this simple setting, given that 
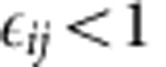
, the Hopf bifurcations should be most common in the presence of an inverted biomass pyramid, typically occurring in planktonic^19^ or other aquatic^20^ systems. This prediction is quite suggestive, because in general it is not possible to predict the type of bifurcation simply looking at basic quantities such as *μ*_U_ and *μ*_L_. Our hypothesis could be investigated both theoretically and empirically.

## Discussion

**Figure 4:**
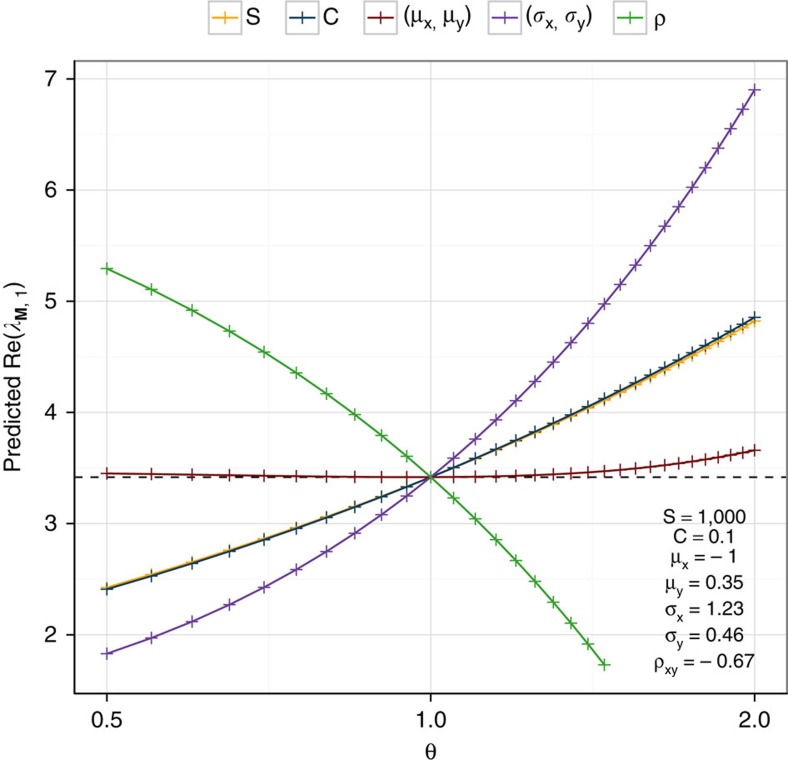
Sensitivity of stability. We can use our approximation to predict Re(*λ*_**M**,1_) for a food web of size *S*, connectance *C* and with strengths sampled from *Z*=(*X*,*Y*), with means *μ*_x_ and *μ*_y_, variances *σ*_x_^2^ and *σ*_y_^2^ and correlation *ρ*_xy_. We predict the change in Re(*λ*_**M**,1_) when any of these parameter is increased (decreased) by multiplying it by the value *θ* (for example, yellow, when *S* is multiplied by *θ*, or purple, when both standard deviations are scaled by *θ*). The effect of making the means *μ*_x_ and *μ*_y_ twice (half) as strong is small, compared with the effect of altering the variances or the correlation. Increasing (decreasing) *S* or *C* yield almost identical effects—consistently with the stability–complexity^21^ relationship. The black dashed line marks the value of Re(*λ*_**M**,1_) for the parameters reported in the Figure (that is, *θ*=1), and the coloured lines represent the increase (decrease) expected when changing the corresponding parameter(s).

The new approximation allows us to quantify the contribution of several key quantities to the stability of large food webs. Take a food web built by the cascade model for a given size *S*, connectance *C* and parameterized using the bivariate distribution *Z*=(*X*,*Y*) defined by its means *μ*_x_ and *μ*_y_, its variances *σ*_x_^2^ and *σ*_y_^2^ and the correlation *ρ*_xy_. In Fig. 4, we show how Re(*λ*_**M**,1_) responds to changes in the parameters, by re-calculating the approximation when a given parameter is multiplied by a factor *θ*.

Interestingly, even doubling (or halving) the average interaction strengths, *μ*_x_ and *μ*_y_ has very little effect on the stability of the system. This is due to the fact that, when −*μ*_U_>*μ*_L_ and Re(*λ*_**A**,1_) is very constrained, and increasing the average strength of interaction simply makes the large eigenvalues with negative real part even more negative, with negligible effects on stability (note, however, that average strengths would be the most important quantities when −*μ*_U_<*μ*_L_; Supplementary Note 3). The size and connectance have a stronger effect (confirming the inverse relationship between stability and ‘complexity'^21^), but far less than the variances and the correlation, with increasing variances being strongly destabilizing, and high negative correlation being strongly stabilizing. These observations question a large body of literature^11^,^12^,^13^,^14^ focusing on the relationship between mean interaction strength and stability.

Here we have derived for the first time an analytic approximation able to predict the stability of large, structured food webs. The approach is based on the decomposition of the community matrix into the sum of two matrices, and the same approach could be used to study the influence of stability of other important food web properties, such as modularity^17^, the presence of trophic groups^22^ and the division into trophic levels.

## Methods

### Distribution of interaction strengths

We build an empirical distribution for *Z* using a large database detailing the relationship between consumer and resource body sizes for thousands of observed trophic interactions^23^. To transform body-size relationships into the coefficients of the community matrix, we need to estimate the interactions between species as well as the equilibrium biomasses for all populations. To this end, we make use of body-size scaling theory^5^ to construct a reasonable distribution *Z* (Supplementary Note 2). All the figures presented here are obtained for a particular choice of parameters, but our results hold also for alternative parameterizations, and even for entirely different distributions (Supplementary Note 4). In particular, the results are consistent with the universality property found for other random matrices^2^,^3^: once fixed the mean and covariance matrix, the details of the distribution of the coefficients have no effect on the distribution of the eigenvalues.

### Spectrum of **A** and **B**

In Supplementary Note 3, we derive the eigenvalues of **A**. All eigenvalues fall on the curve describing a circle in the complex plane with


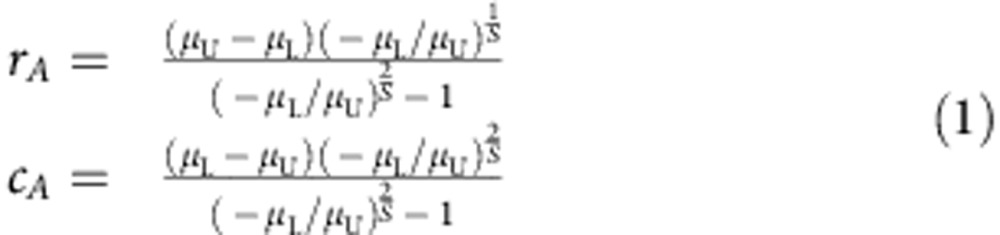


where *r*_*A*_ is the radius of the circle, and is (*c*_*A*_,0) its center.

For matrix **B**, we conjecture that its eigenvalues are approximately uniformly distributed in the ellipse in the complex plane with horizontal semi-axis *r*_*h*,*B*_≈(*α*+*ρ*_UL_*σ*_U_*σ*_L_(*S*−1))/(*α*)^1/2^ and vertical semi-axis *r*_*v*,*B*_≈(*α*−*ρ*_UL_*σ*_U_*σ*_L_(*S*−1))/(*α*)^1/2^, where *α*/*S* tends to (*σ*_U_^2^−*σ*_L_^2^)/log(*σ*_U_^2^/*σ*_L_^2^) for *S* large (Supplementary Note 3). In the limit of *σ*_U_→*σ*_L_, we obtain *α*≈*S*, consistently with the elliptic law.

## Additional information

**How to cite this article:** Allesina, S. *et al.* Predicting the stability of large, structured food webs. *Nat. Commun.* 6:7842 doi: 10.1038/ncomms8842 (2015).

## Supplementary Material

Supplementary Information Supplementary Figures 1-27, Supplementary Notes 1-5 and Supplementary References
