# Supplementary material for: Predicting the stability of large structured food webs
Source: Nat Commun. 2015 Jul 22;6:7842. doi: 10.1038/ncomms8842 (PMC4525179; doi:10.1038/ncomms8842)
Supplement: Supplementary Information — Supplementary Figures 1-27, Supplementary Notes 1-5 and Supplementary References [file ncomms8842-s1.pdf]

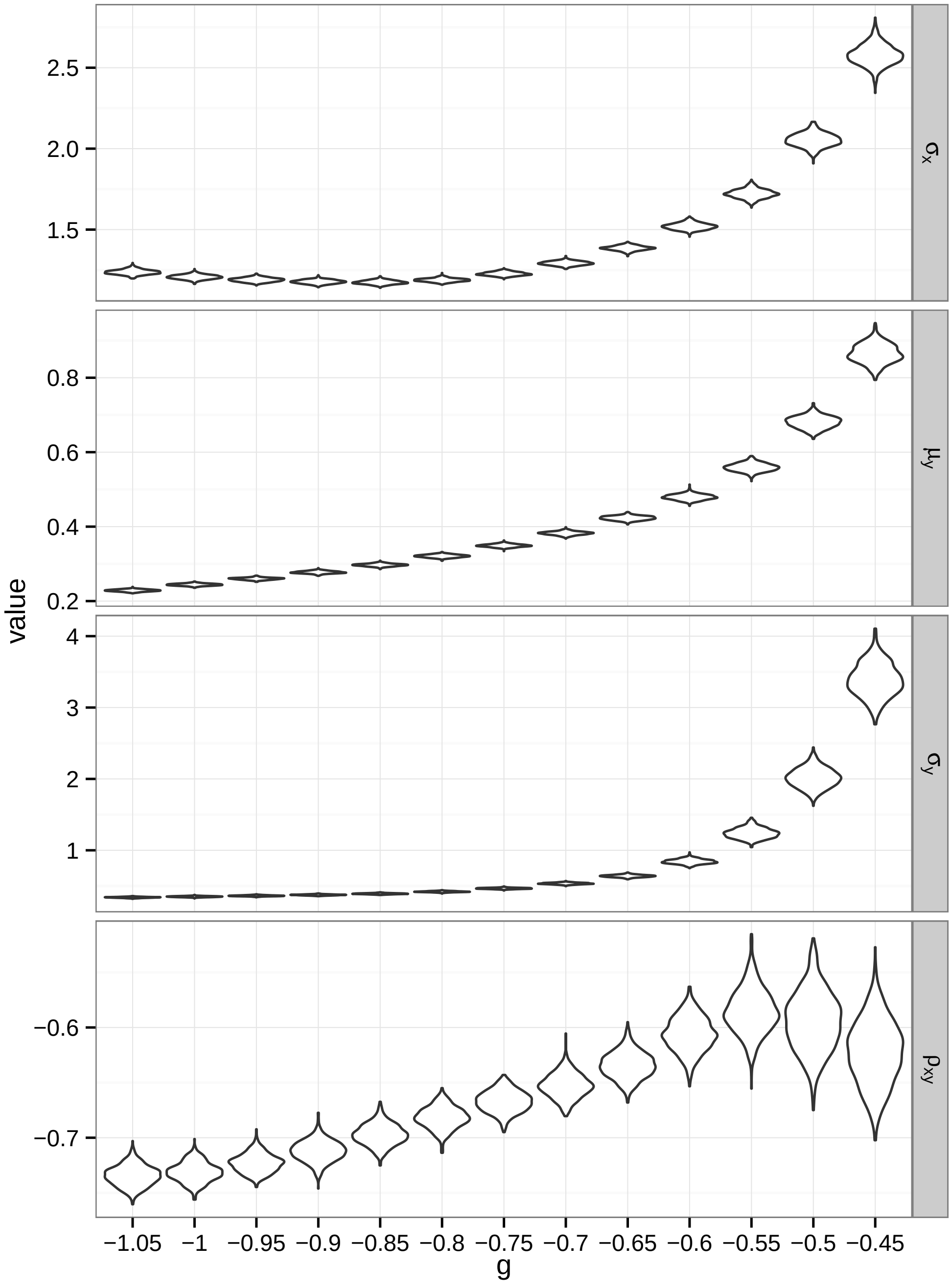

Supplementary Figure 1: *Violin plots showing the effect of g, the main parameter determining the scaling between the body mass and equilibrium abundance, on: i)* σ_x*, the standard deviation of the negative effects (effect of consumer on resource); ii)* μ_y*, the mean of the positive effects (resource on consumer); iii)* σ_y*, the standard deviation of the positive effects; iv)* ρ_{xy}*, the correlation between positive and negative effects. The coefficients are rescaled to obtain* $\mu_x = -1$ *(for ease of comparison).*

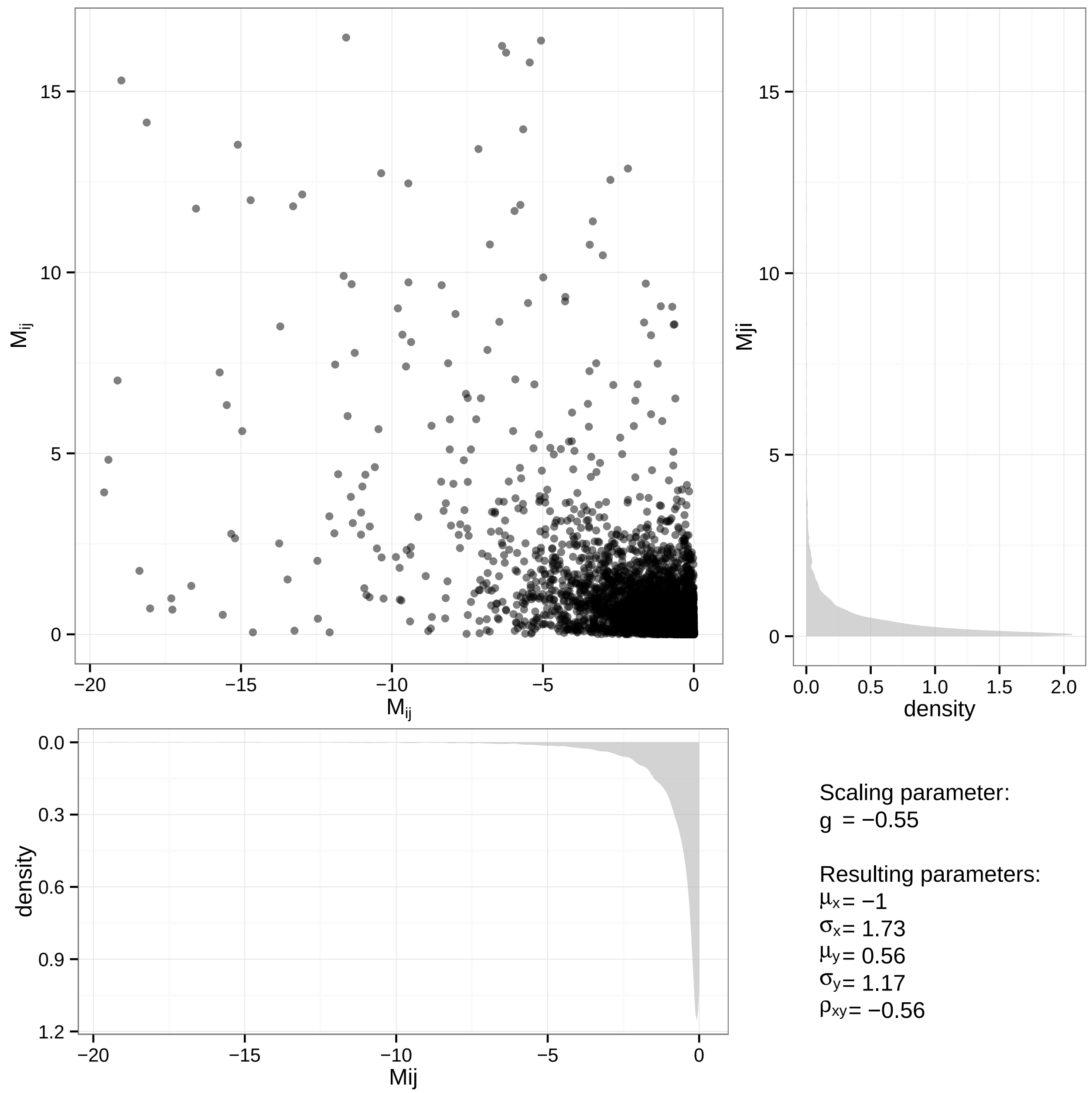

Supplementary Figure 2: *Distribution of M_{ij} (negative effects, x-axis) and M_{ji} (positive, y-axis) obtained as specified in the text when $g = -0.55$. The two marginal distributions have similar shapes, with high skeweness and few large values.*

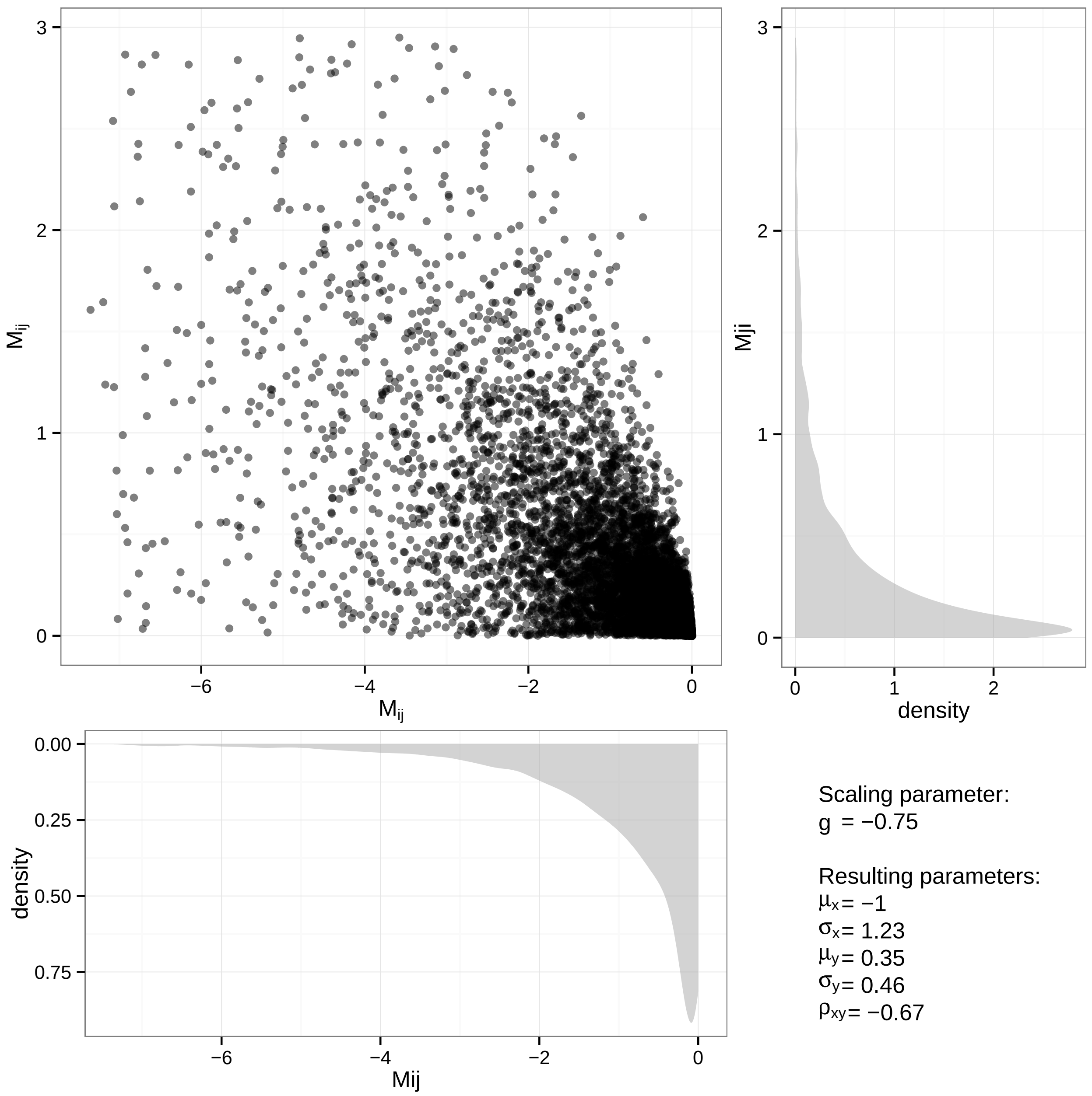

Supplementary Figure 3: *As Figure 2, but with* $g = -0.75$.

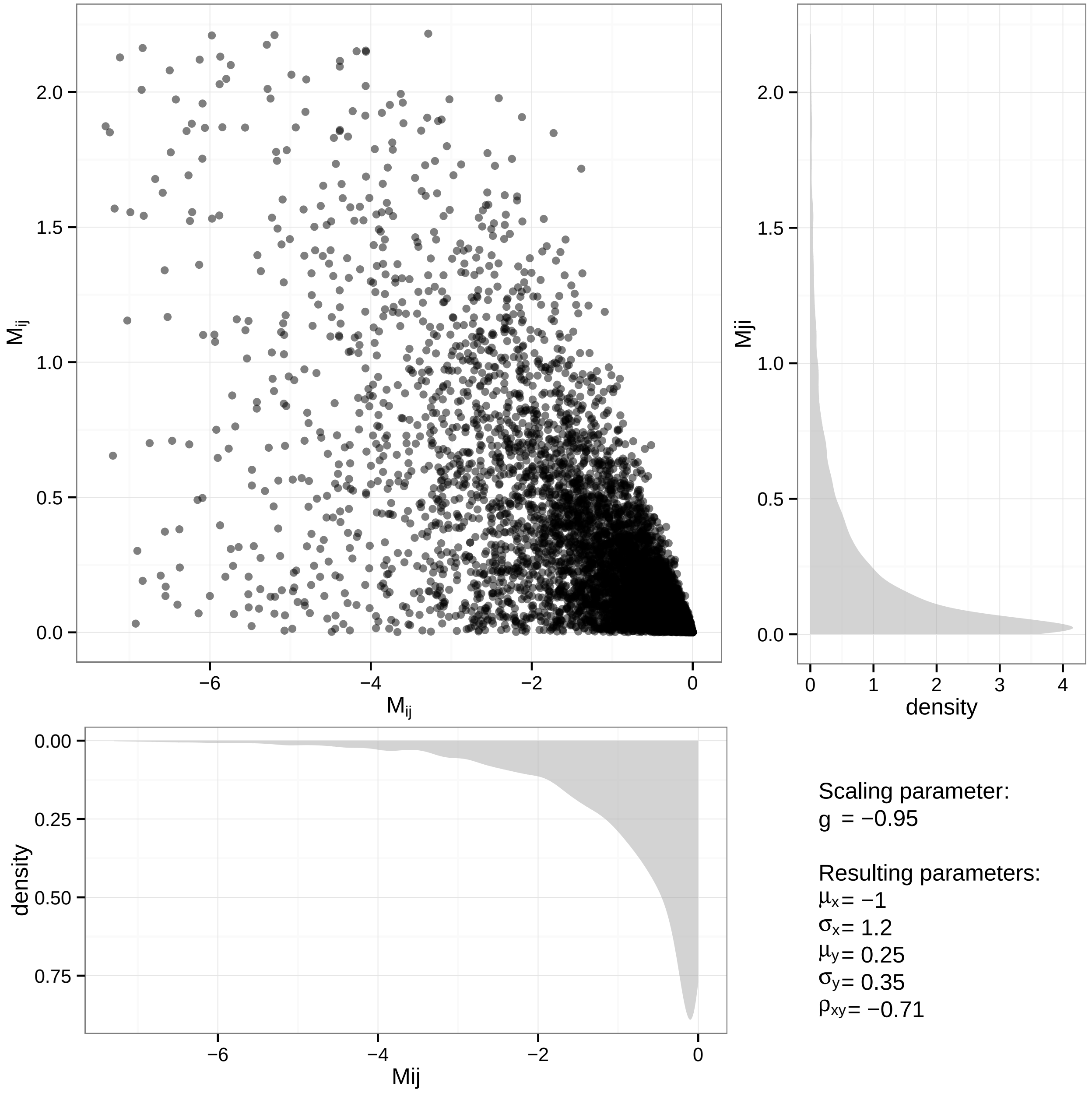

Supplementary Figure 4: *As Figure 2, but with* $g = -0.95$.

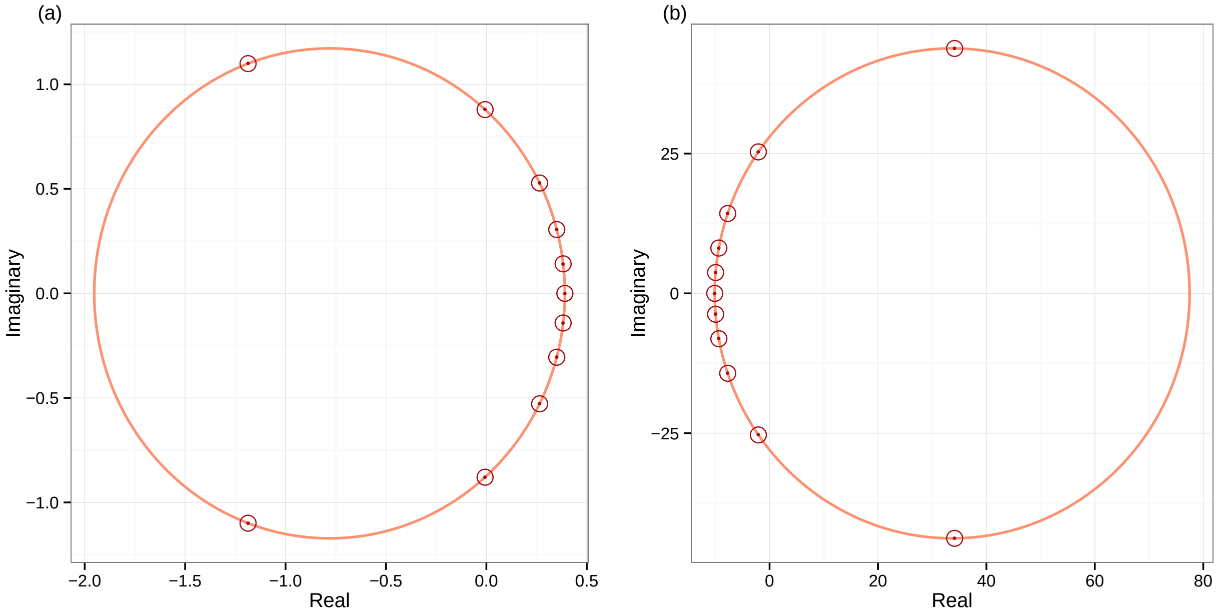

Supplementary Figure 5: *Spectrum of* **A***, a matrix constructed such that the diagonal is* 0*, all lower-triangular coefficients are equal to x and all upper-triangular coefficients are* -1*. (a) The case when $S = 11$ and $x = 0.01$. (b) $S = 11$ and $x = 25$. The dots represent the solution obtained by numerical diagonalization; the red circles the prediction following Eq. 27, and the solid line the predicted curve* where the eigenvalues fall.

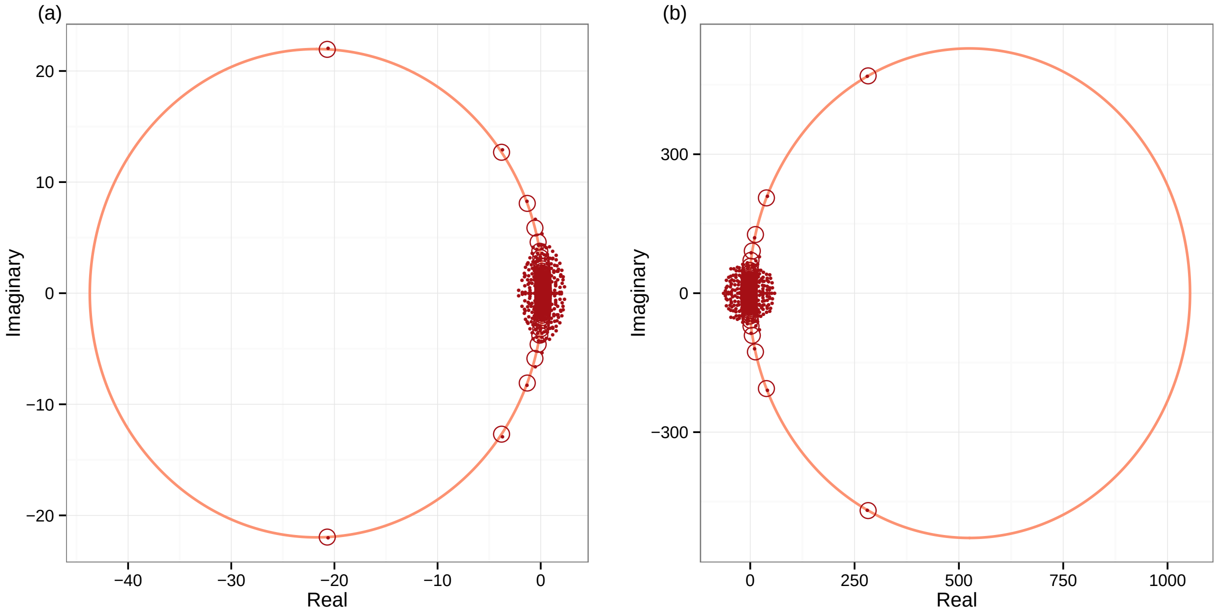

Supplementary Figure 6: *(a) Spectrum of M, a matrix of size* 250 *constructed such that the diagonal is* 0*, while the off-diagonal pairs* $(M_{ij}, M_{ji})_{j>i}$ *are set to* $(0,0)$ *with probability* $(1-C)$ *and to* $(-1, 0.05)$ *with probability* $C = 0.5$*. (b) Spectrum of M, a matrix of size* 250 *constructed such that the diagonal is* 0*, the upper-triangular coefficients are independently sampled from a Normal distribution with mean* $\mu_U = -1$ *and variance* ${\sigma_U}^2 = 16$*, while the lower-triangular are sampled independently from a Normal distribution with mean* $\mu_L = 7$ *and variance* ${\sigma_L}^2 = 16$*. The red circles represent the predictions for the eigenvalues of a deterministic matrix* $\mathbf{A}$ *with* μ_U *above the diagonal and* μ_L *below the diagonal, obtained using Eq. 27. While the eigenvalues with large modulus are accurately predicted by the formula, those close to zero are not.*

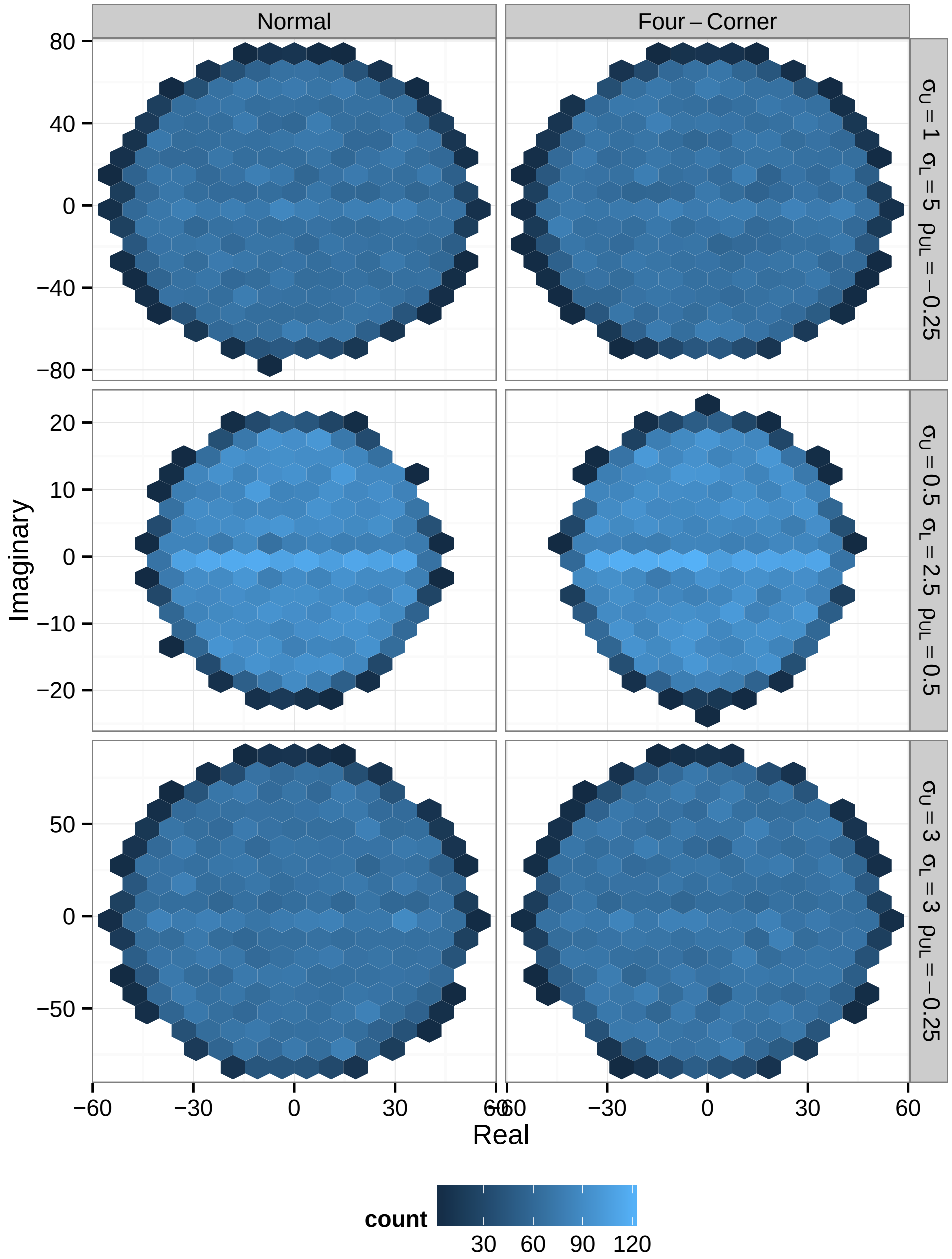

Supplementary Figure 7: *Illustration of assumption a). For any choice of parameters σ_{U}, σ_{L}, ρ_{UL}, the eigenvalues of* **B** *are approximately uniformly distributed in an ellipse. We plot the number of the eigenvalues in each hexagon obtained by pooling the eigenvalues of* 25 *matrices of size* 500×500*, built using the parameters specified for each panel and two different distributions. Irrespective of the choice of parameters, we observe approximately uniform density in an ellipse, as we would expect for the case of equal variances (bottom panels).*

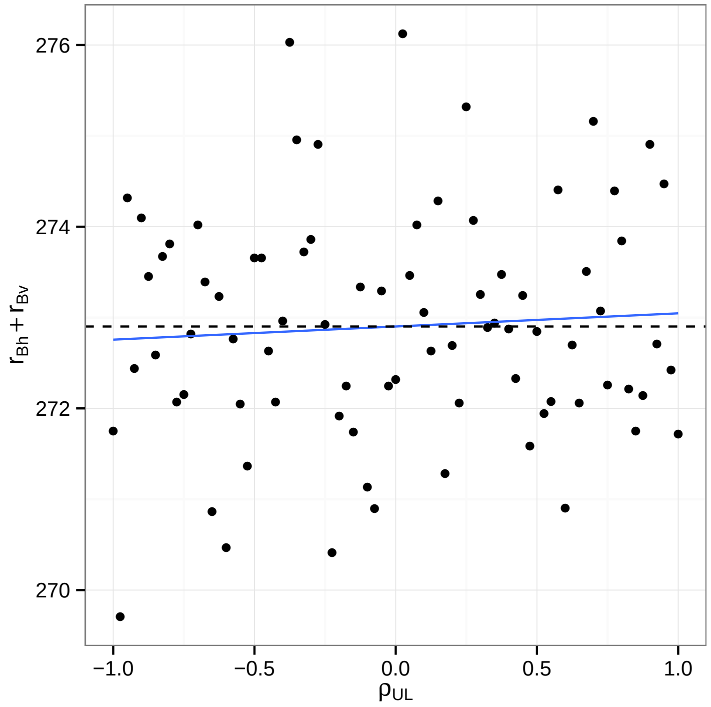

Supplementary Figure 8: *Illustration of assumption b). For any choice of parameters σ_U and σ_L, the sum of the horizontal and vertical semi-axes of the ellipse containing the eigenvalues is approximately constant. Because the actual value of the semi-axes is unknown, we plot the sum of the maximum real and maximum imaginary parts of the eigenvalues of a single matrix* **B***, for each choice of ρ_{UL}. The coefficients of* **B** *(of size $S = 2500$) are sampled from a bivariate Normal distribution with $\sigma_U = 1$, $\sigma_L = 5$, and correlation ρ_{UL} (x-axis). The blue line is the best-fitting line, and the dashed black line marks the mean of the observed values.*

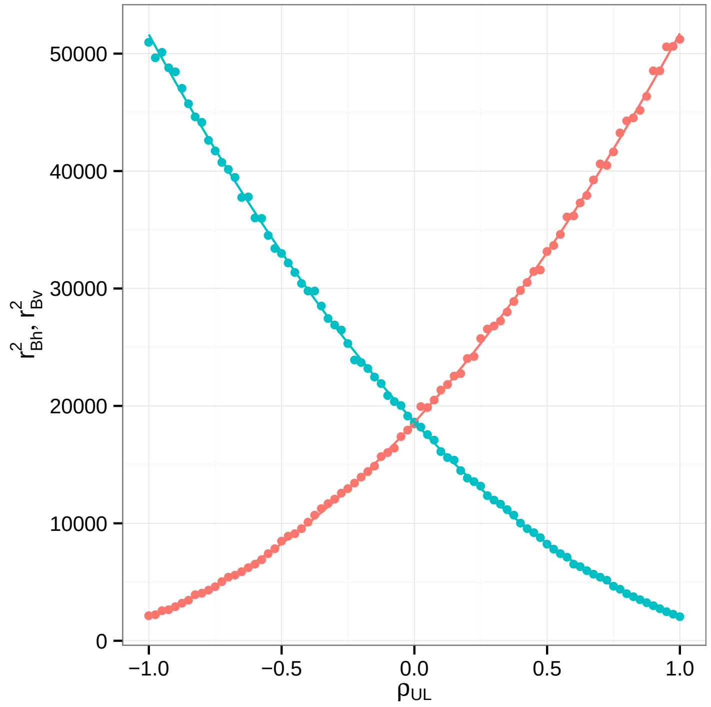

Supplementary Figure 9: *Illustration of assumption c). For any choice of parameters* σ_U *and* σ_L, $r^2_{B,h}$ *(red) and* $r^2_{B,v}$ *(blue) are well described by a second-degree polynomial (lines) in* ρ_{UL} *(x-axis). Moreover,* $r_{B,h}$ *when the correlation is* ρ_{UL} *is equal to* $r_{B,v}$ *when the correlation* $-\rho_{UL}$*, producing a symmetry about 0. The matrices are the same as in Figure 8.*

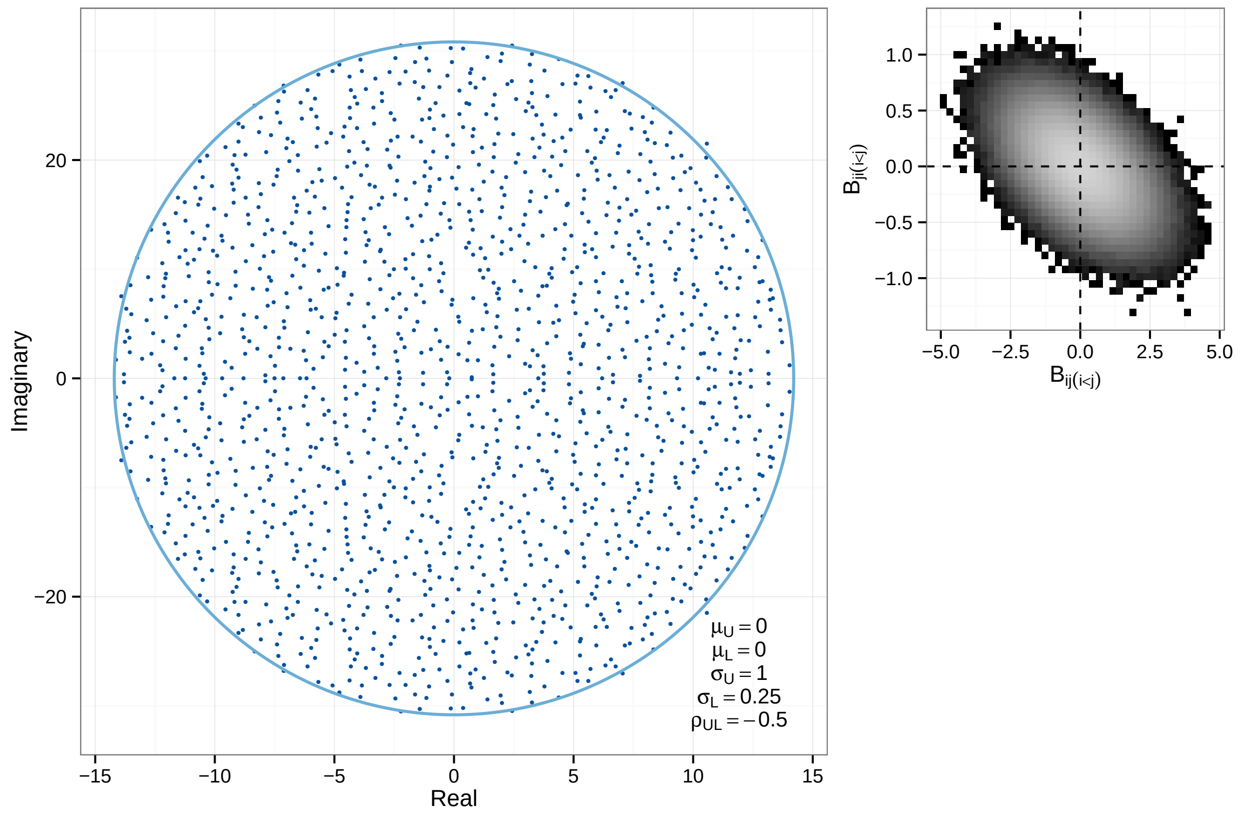

Supplementary Figure 10: *Spectrum of a single* 1500×1500 *matrix* $\mathbf{B}$, *where the coefficients* (B_{ij}, B_{ji}) *are sampled in pairs from a bivariate Normal distribution with* $\sigma_U = 1$, $\sigma_L = 1/4$ *and correlation* $\rho_{UL} = -1/2$. *The distribution of the coefficients is drawn in the top-right panel. The semi-axes of the light blue ellipse are calculated using Eq. 44.*

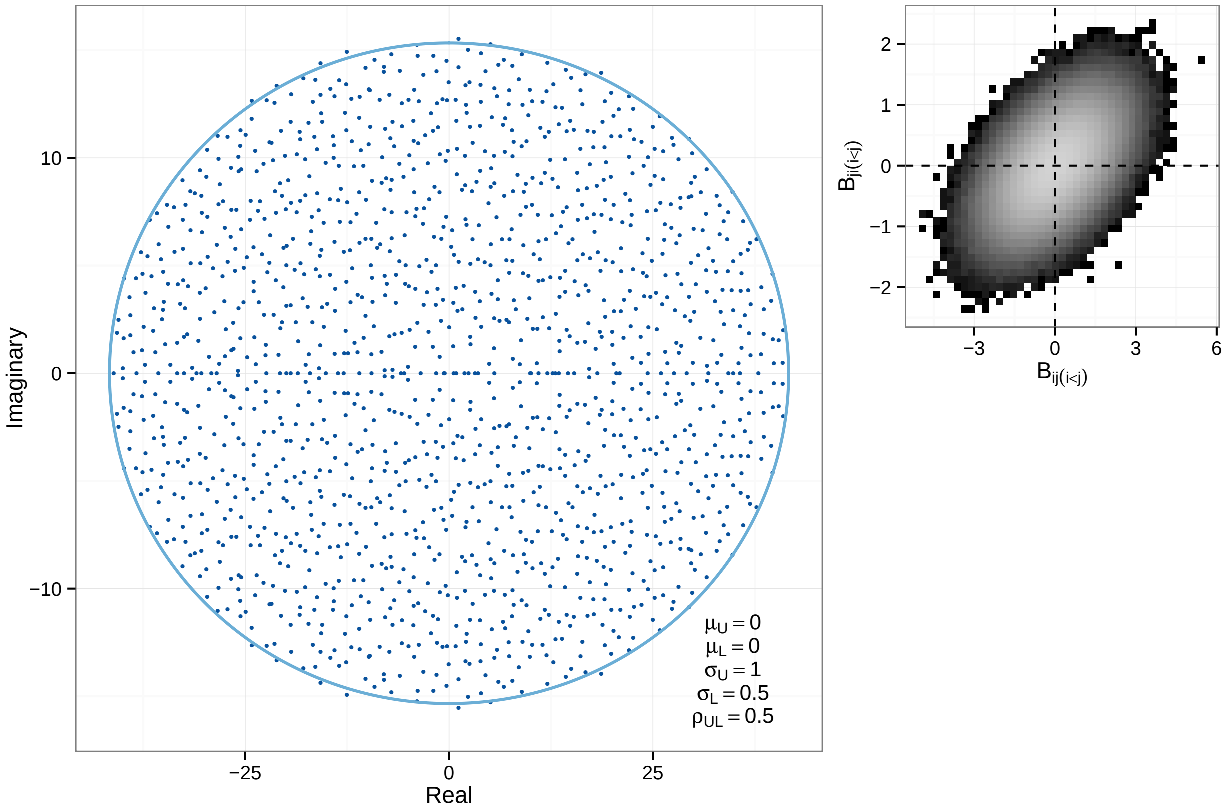

Supplementary Figure 11: *As Figure 10, but with* $\sigma_L = 1/2$ *and correlation* $\rho_{UL} = 1/2$.

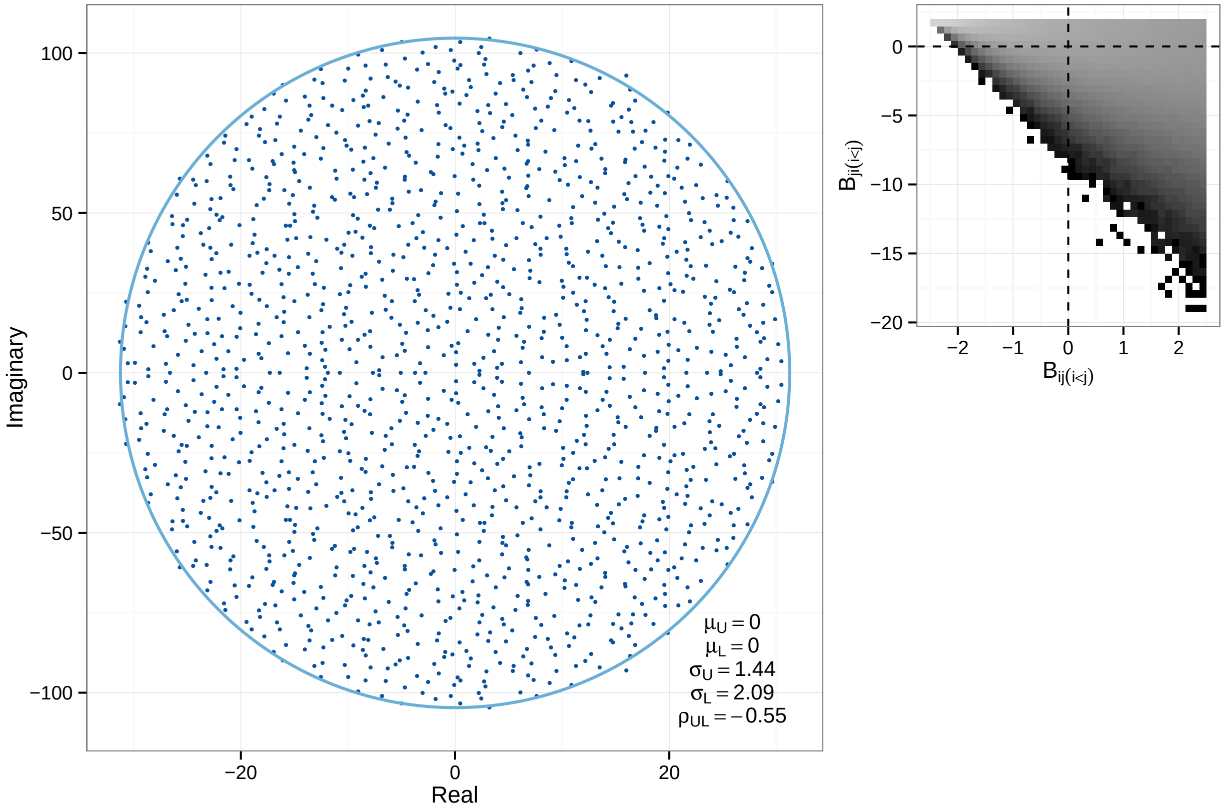

Supplementary Figure 12: *As in Figure 10, but with non-Normal marginals. We sampled each* B_{ij}*, with* $i < j$ *from a Uniform distribution* $\mathcal{U}[0,5]$*, while the corresponding* B_{ji} *was obtained multiplying* B_{ij} *for a randomly sampled number from the negative standard Half-Normal distribution. The means of the pairs were made zero by subtracting the corresponding means from the upper- and lower-triangular parts of the matrix.*

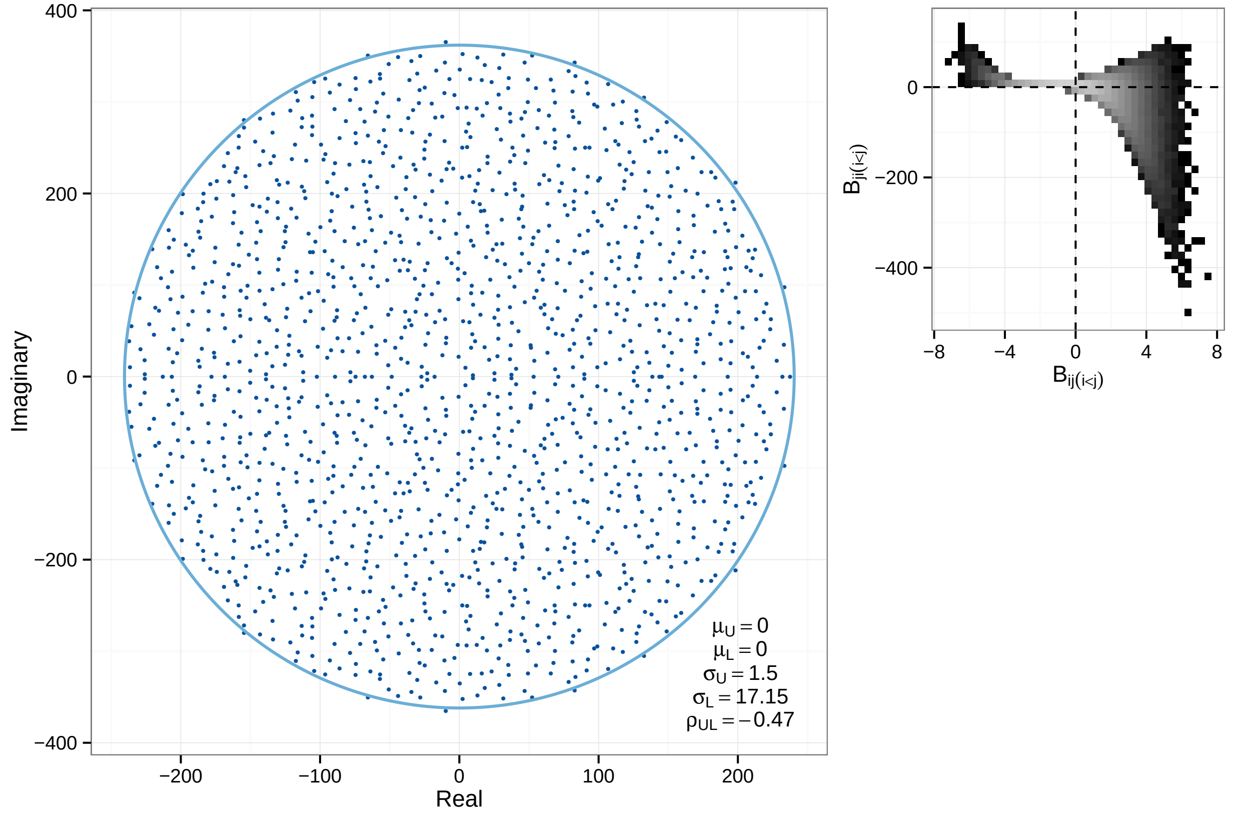

Supplementary Figure 13: *As in Figure 12, but with a more complicated distribution for the coefficients.*

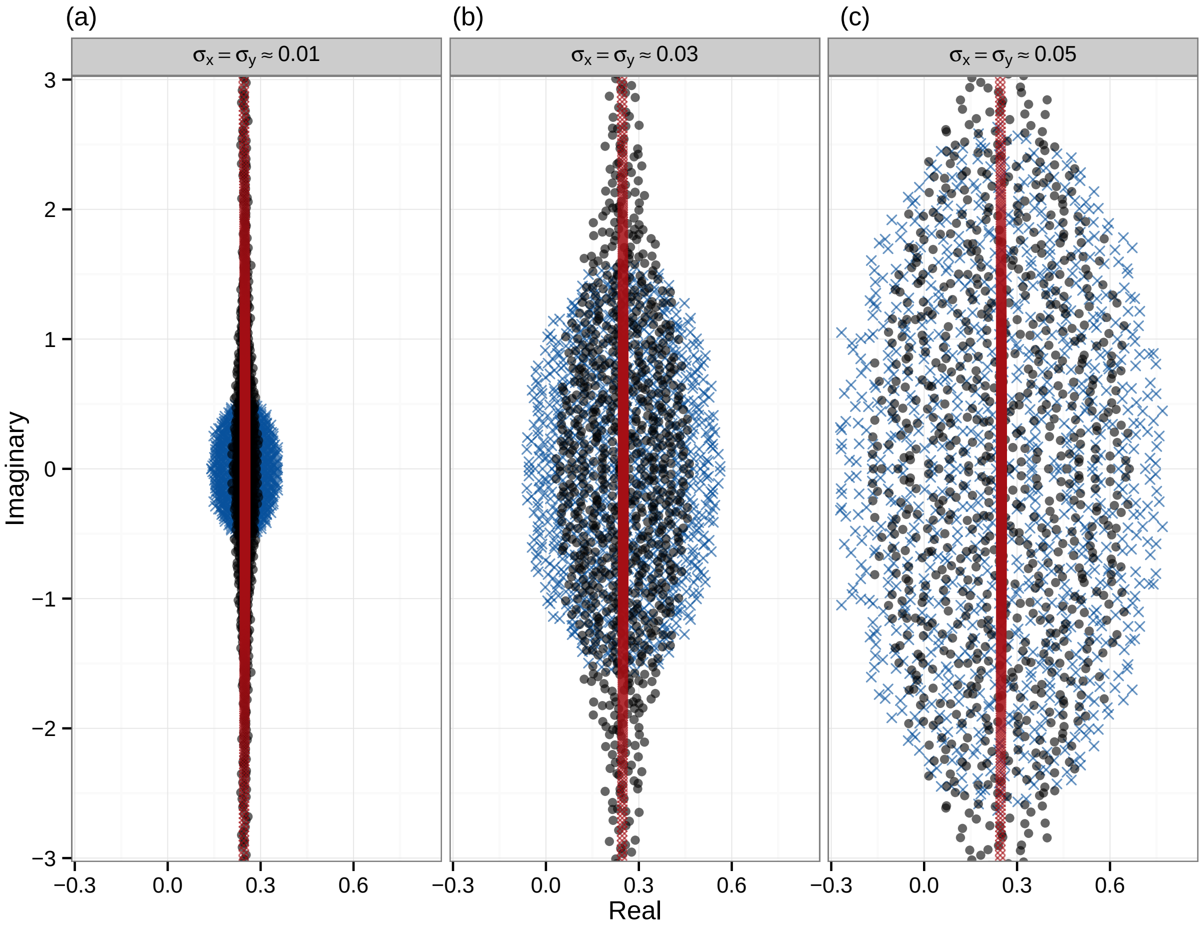

Supplementary Figure 14: *Eigenvalues of the matrices* **M** *(black),* **A** *(red), and* **B** *(blue, shifted to the right by* $Re(\lambda_{\mathbf{A},1})$*) built with* $S = 1000$, $C = 1$, *and coefficients sampled from a bivariate Normal with* $\mu_x = -1$, $\mu_y = 1/2$, *and* $\rho_{xy} = -2/3$. *(a) When* $\sigma_x = \sigma_y \approx 0.01$, *the eigenvalues of* **M** *are not well described using our approximation strategy, as the value* $Re(\lambda_{\mathbf{A},1}) + Re(\lambda_{\mathbf{B},1})$ *grossly overestimates* $Re(\lambda_{\mathbf{M},1})$. *(b) Increasing the variances such that* $\sigma_x = \sigma_y \approx 0.025$ *makes the approximation more accurate. (c) For* $\sigma_x = \sigma_y \approx 0.05$ *the discrepancy is further reduced. In all the panels, only the bulk of the eigenvalues are plotted, as the approximation is always accurate for the eigenvalues with large modulus.*

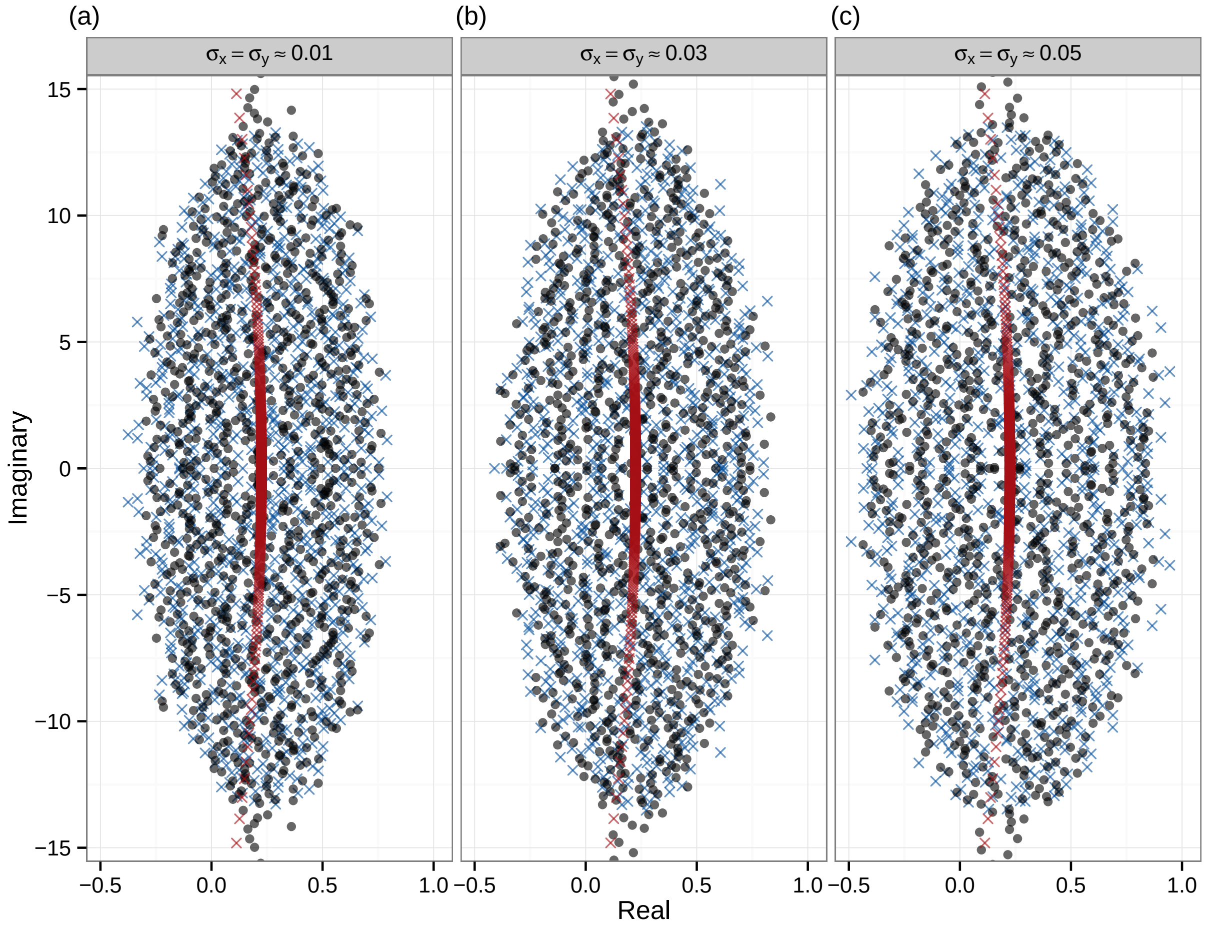

Supplementary Figure 15: *As Figure 14, but with connectance C = 0.9. In this case, a smaller connectance results in larger variances (compared to the* C = 1 *case) of the upper- and lower-triangular part of the matrix* **M**, *leading to very accurate predictions.*

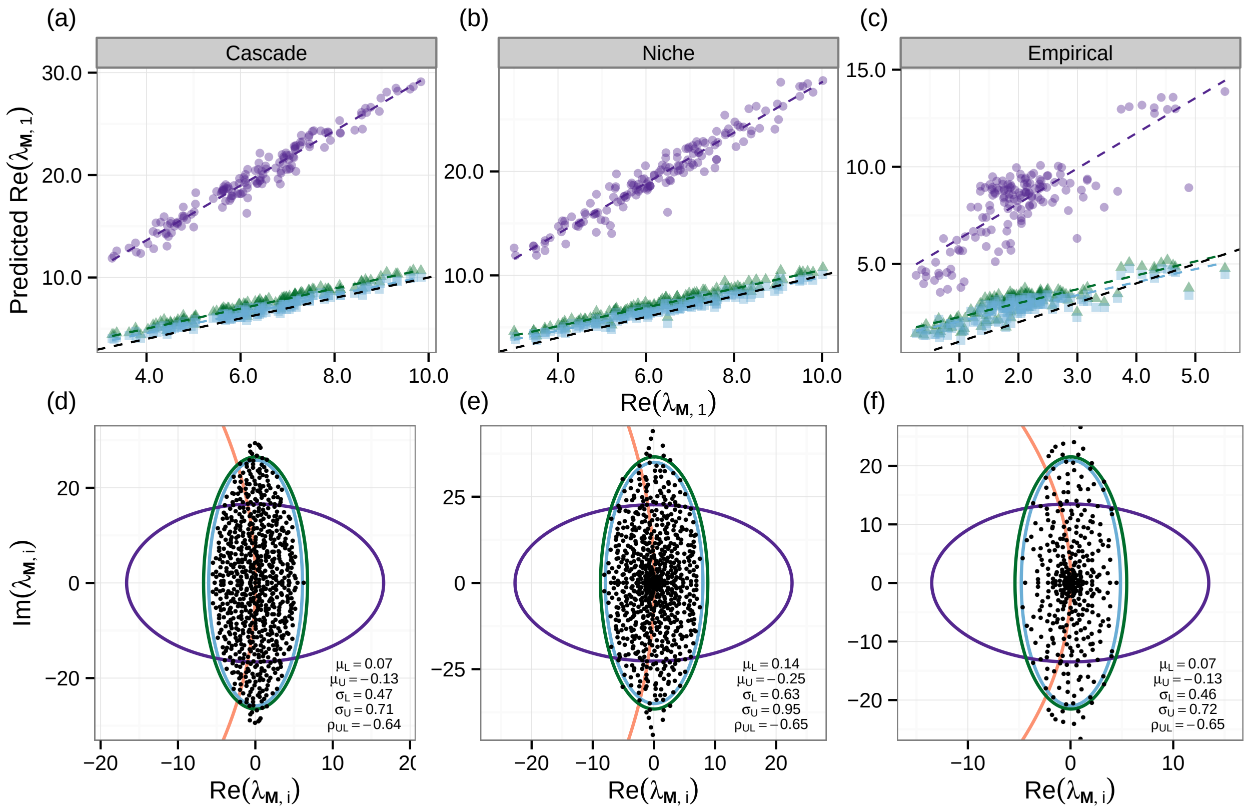

Supplementary Figure 16: *As in Figure 2 of the main text, but using* Z_{55}*. For this parameterization, the results obtained using the approximation of Tang et al.*[1] *and the new approximation are very similar, as both* $-\mu_U \approx \mu_L$*, and* $\sigma_U \approx \sigma_L$*.*

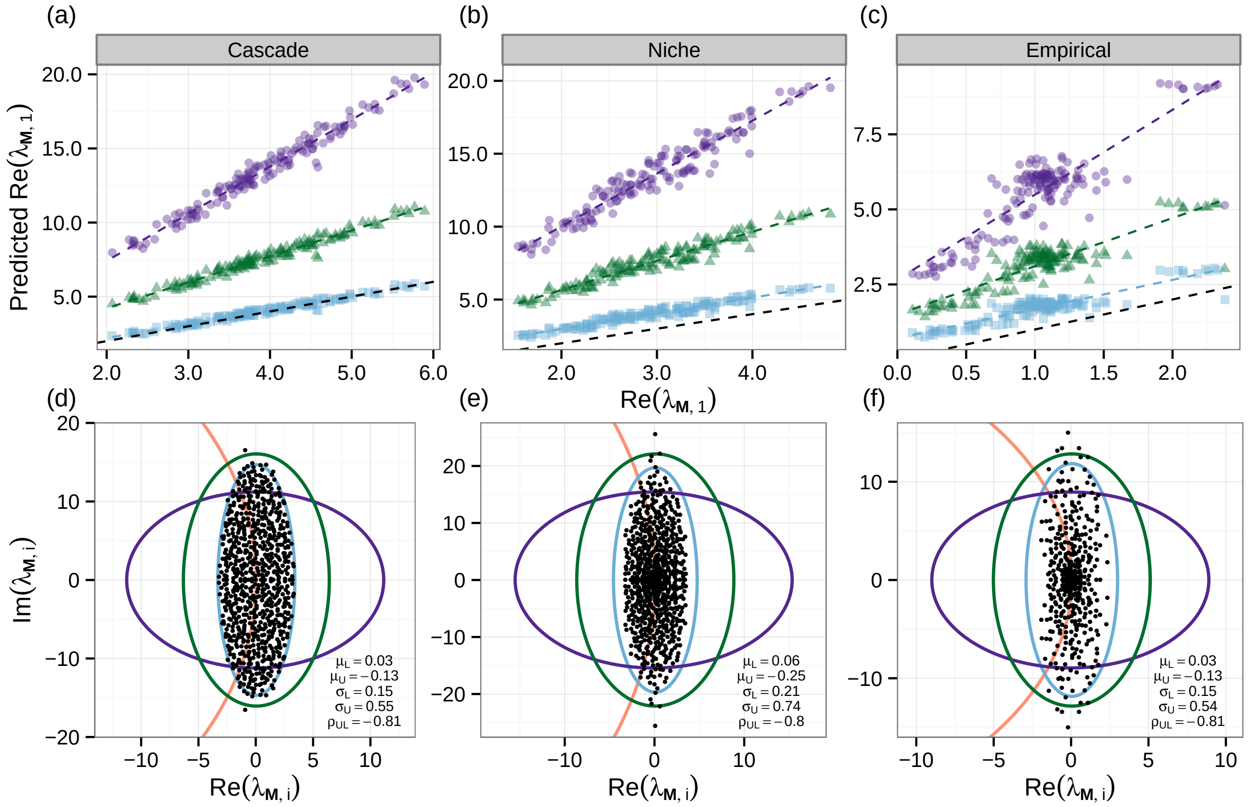

Supplementary Figure 17: *As in Figure 2 of the main text, but using* Z_{95}.

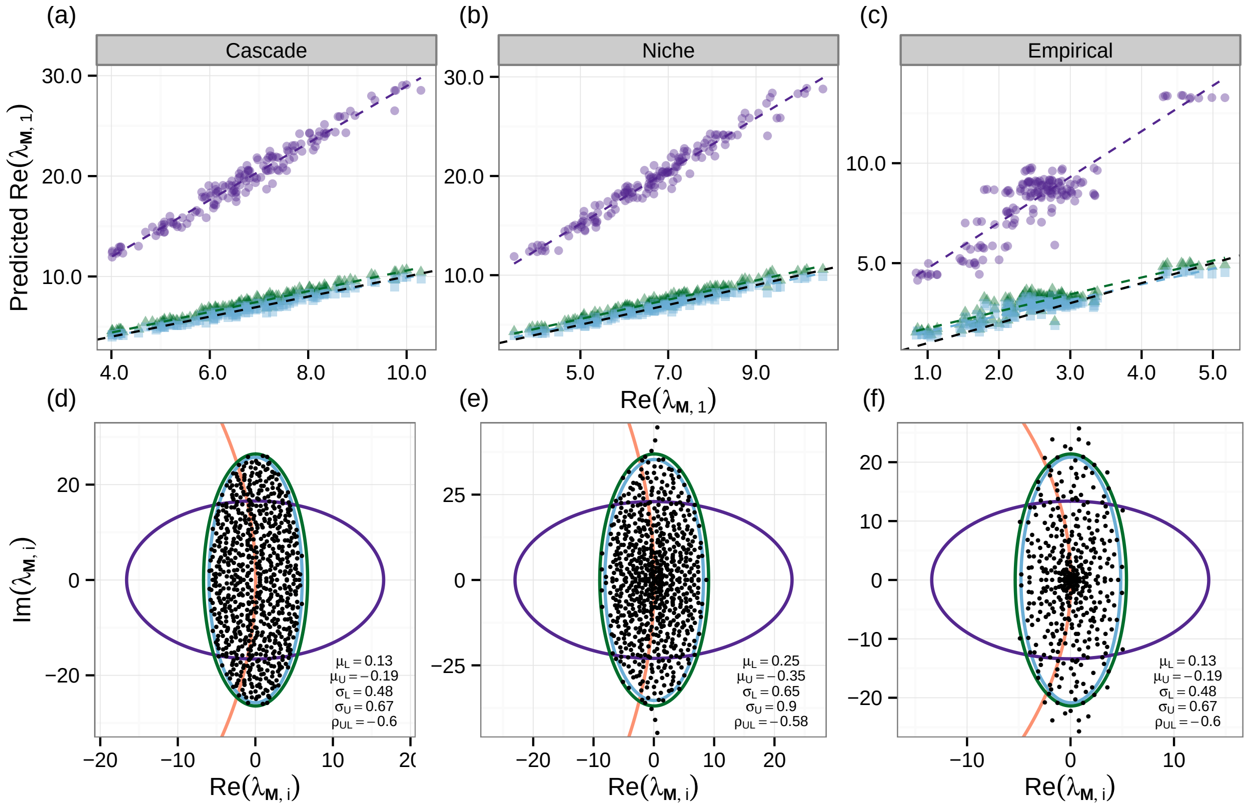

Supplementary Figure 18: *As in Figure 16, but parameterizing the matrices using a bivariate Normal distribution with the same mean and covariance matrix of* Z_{55}.

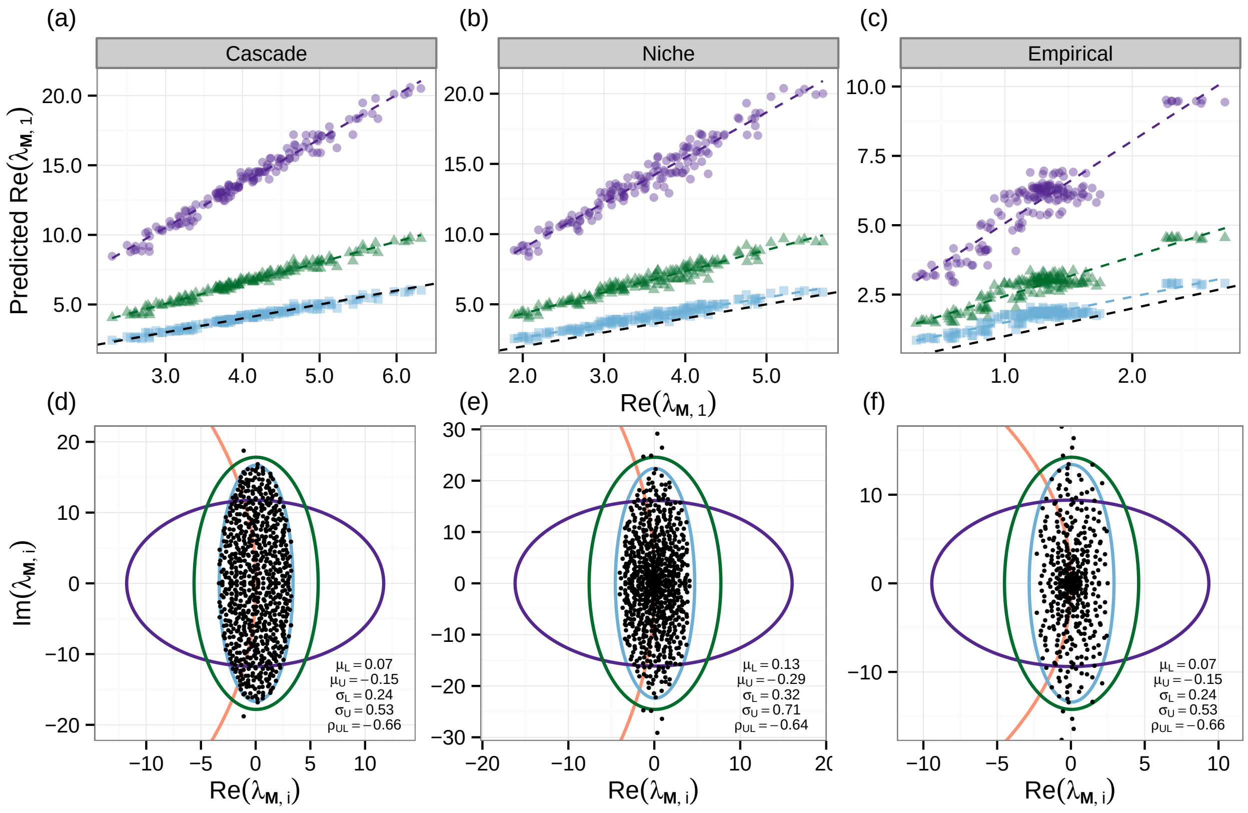

Supplementary Figure 19: *As in Figure 2 of the main text, but parameterizing the matrices using a bivariate Normal distribution with the same mean and covariance matrix of* Z_{75}.

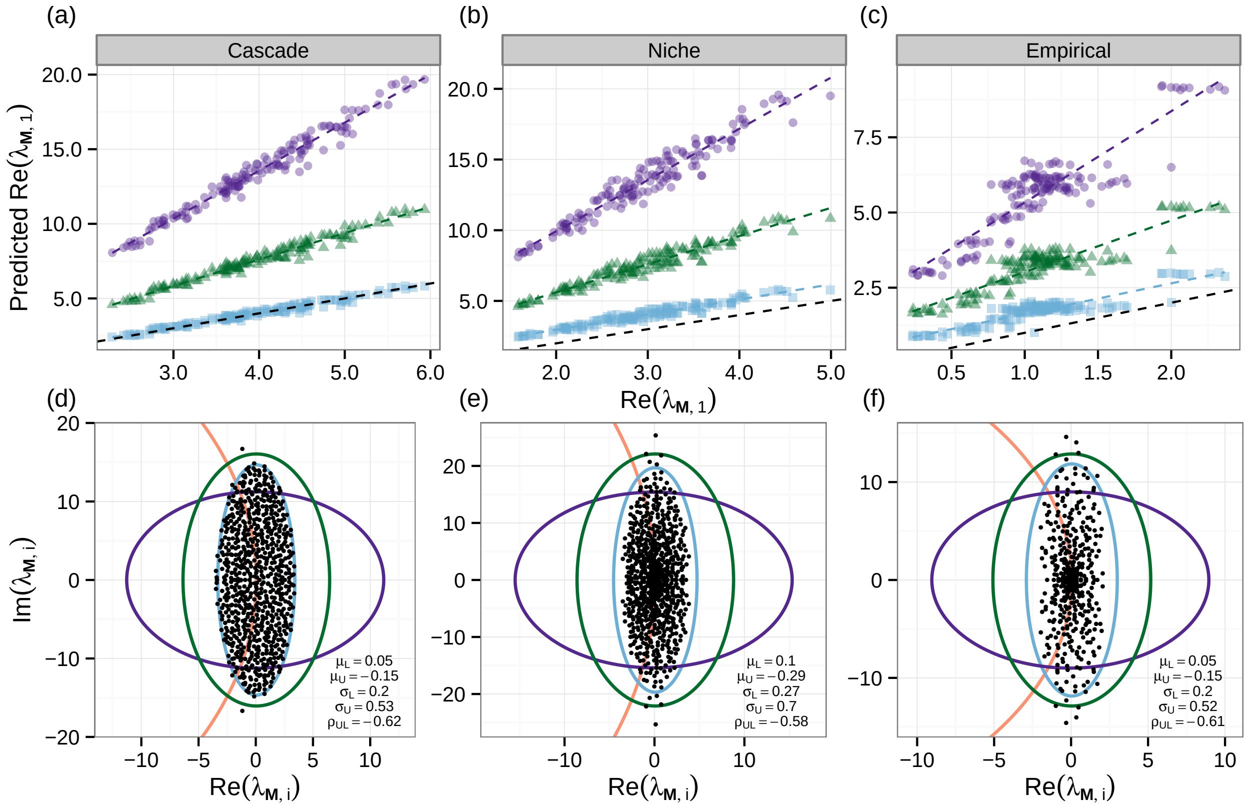

Supplementary Figure 20: *As in Figure 17, but parameterizing the matrices using a bivariate Normal distribution with the same mean and covariance matrix of* Z_{95}.

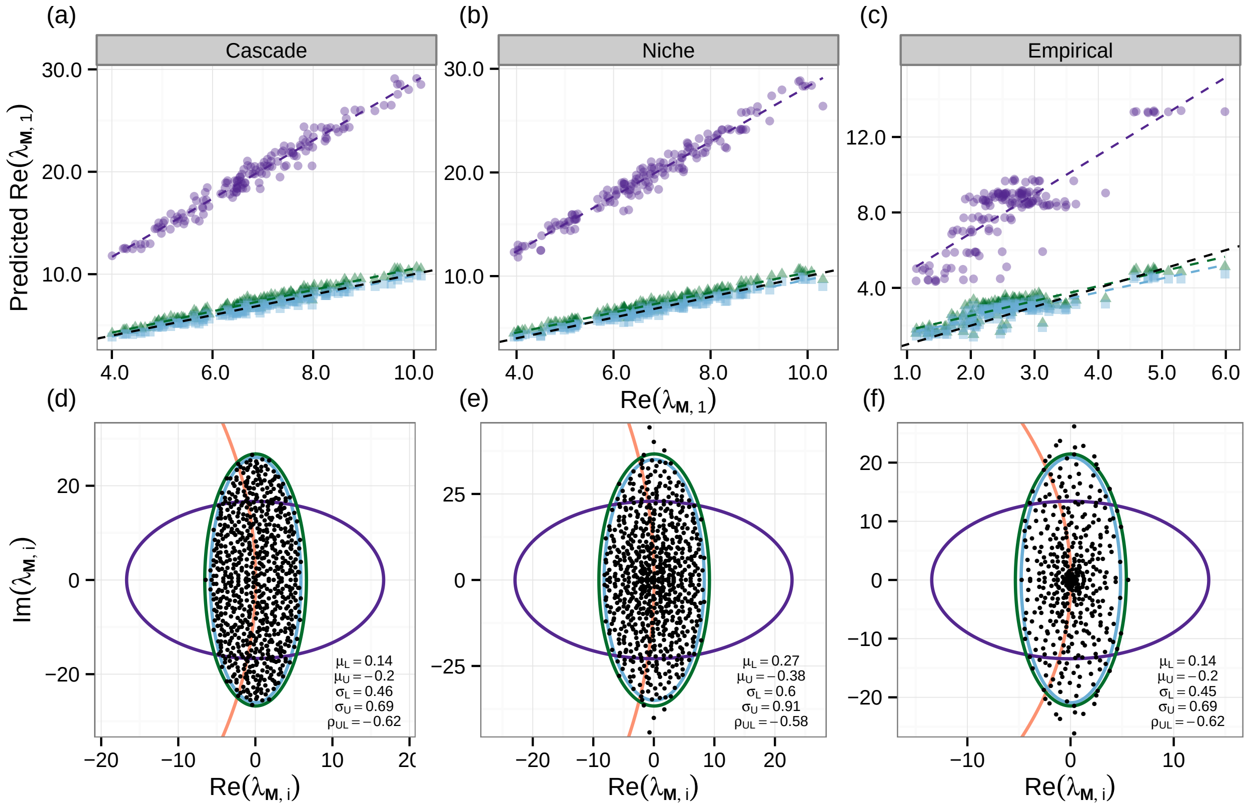

Supplementary Figure 21: *As in Figure 16, but parameterizing the matrices using the Four-corner distribution with the same mean and covariance matrix of* Z_{55}.

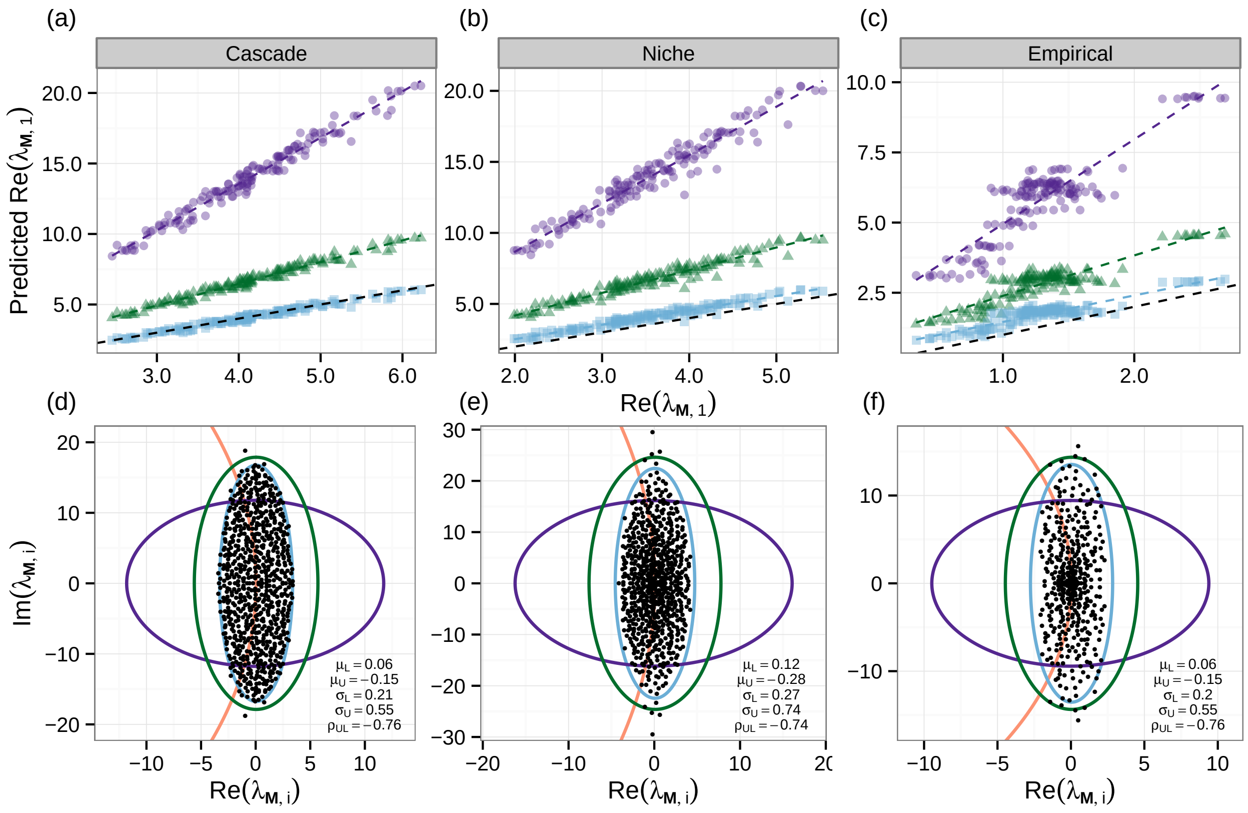

Supplementary Figure 22: *As in Figure 2 of the main text, but parameterizing the matrices using the Four-corner distribution with the same mean and covariance matrix of* Z_{75}.

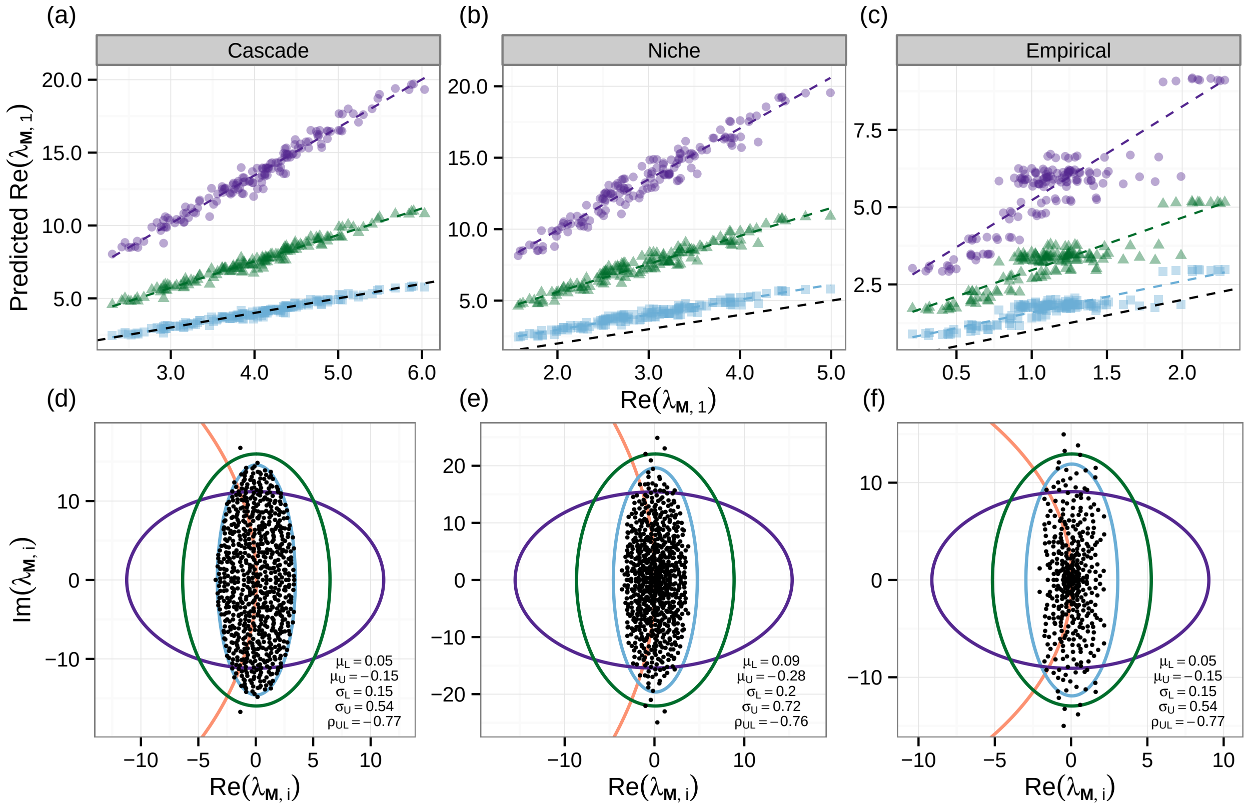

Supplementary Figure 23: *As in Figure 17, but parameterizing the matrices using the Four-corner distribution with the same mean and covariance matrix of* Z_{95}.

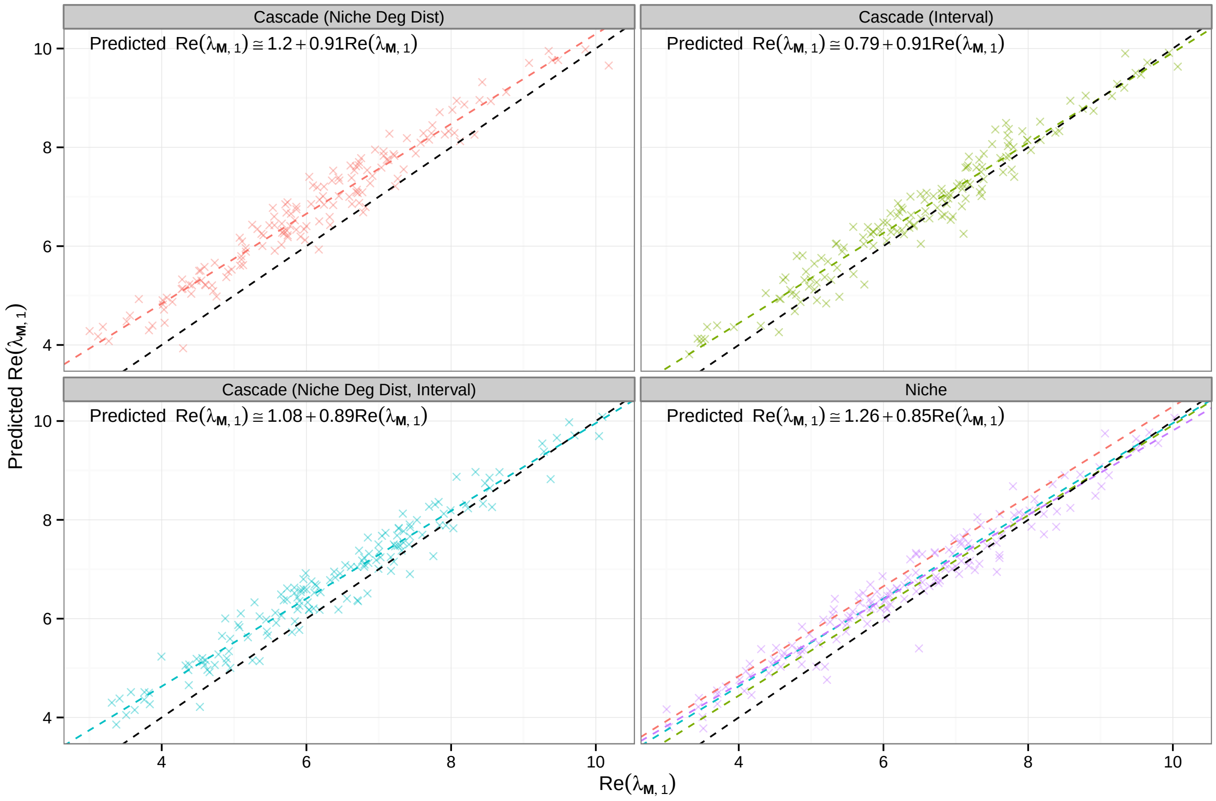

Supplementary Figure 24: *As in Figure 3 of the main text, but parameterizing the matrices using* Z_{55}*.*

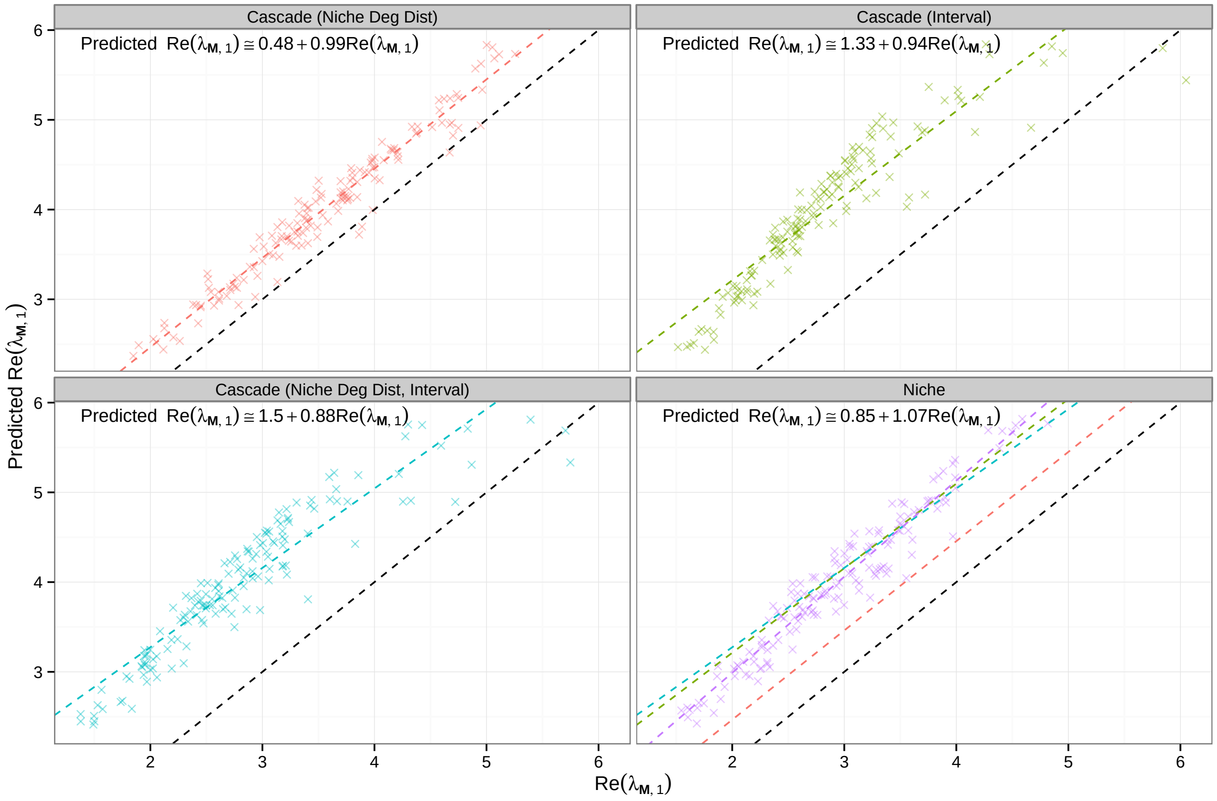

Supplementary Figure 25: *As in Figure 3 of the main text, but parameterizing the matrices using Z_{95}.*

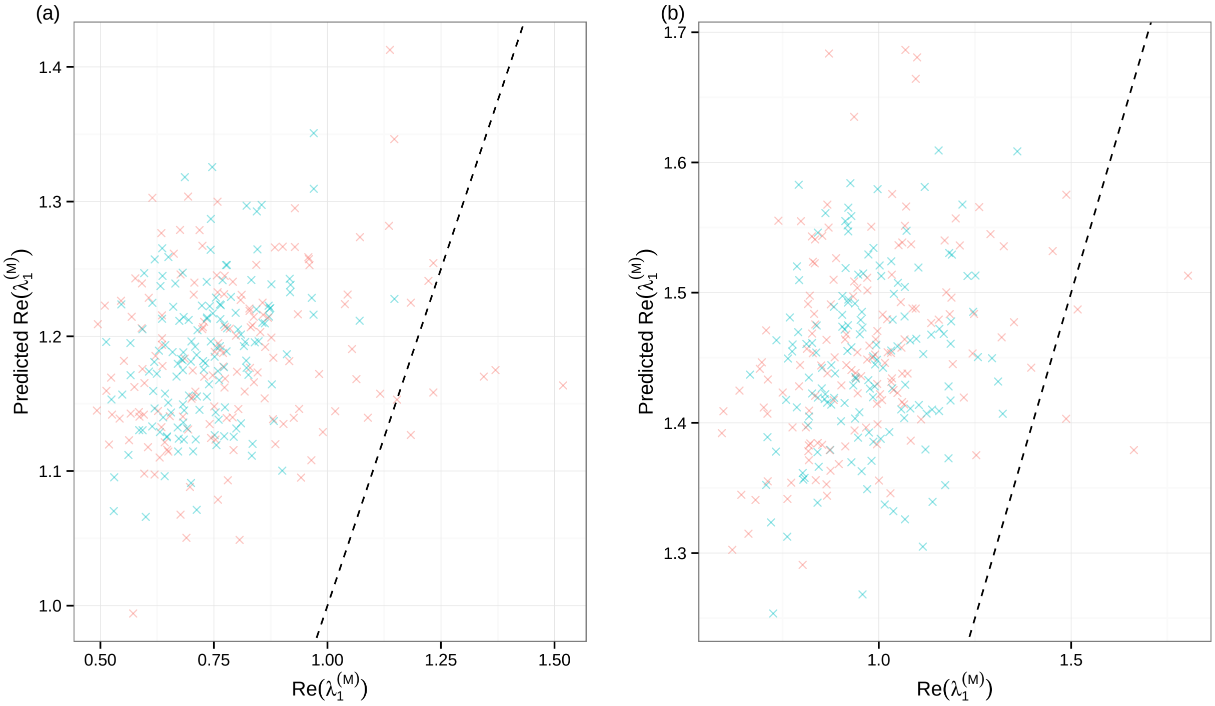

Supplementary Figure 26: *Observed (x-axis) and predicted (y-axis) Re* $(\lambda_{\mathbf{M},1})$ *for 150 parameterizations of an empirical food web (red crosses, (a) Kongs Fjorden, (b) Flensburg Fjord), and 150 parameterizations of a variant of the cascade model preserving the empirical degree distribution (blue crosses). In both cases, conserving the degree distribution produces matrices with very similar leading eigenvalues. All parameterizations produced using* Z_{75}*. Different choices of webs or parameterizations do not alter qualitatively the results.*

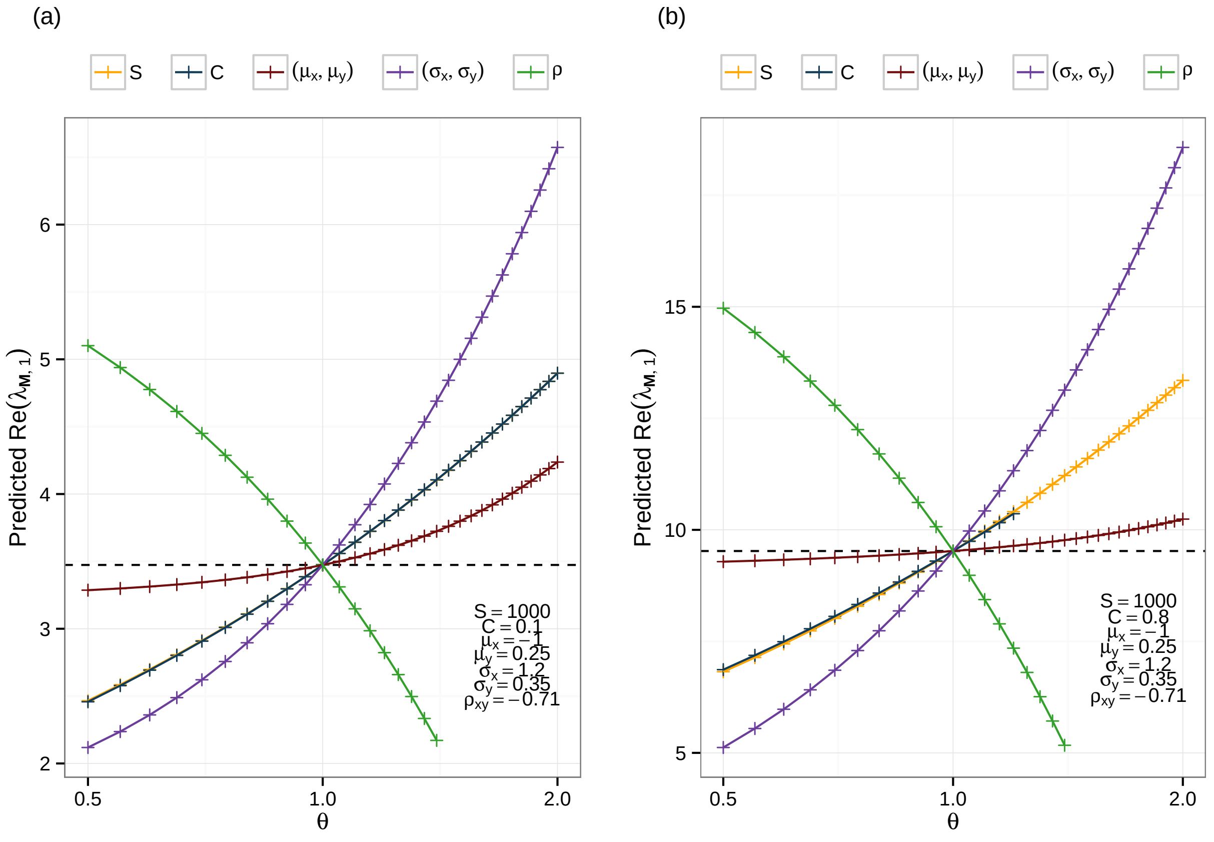

Supplementary Figure 27: *As Figure 4 in the main text, but with parameters taken from* Z_{95} *(reported on the panels). (a) Connectance* $C = 0.1$*. (b)* $C = 0.8$*. For higher connectance, the effect of changing* μ_x *and* μ_y *on stability is more muted.*

Supplementary Note 1: Preliminaries

Here we briefly review local asymptotic stability (henceforth, stability), and two of the main results of Random Matrix Theory. For a more in-depth review of this material, see Allesina & Tang[2].

Stability. We consider a system of autonomous, non-linear ordinary differential equations

$$\frac{dN_i(t)}{dt} = f_i\left(\mathbf{N}(t)\right) \tag{1}$$

where $N_i(t)$ expresses, for example, the biomass of population i at time t, and f_i is the function expressing the growth rate of population i, which depends on the density of all populations ($\mathbf{N}(t)$). The point $\mathbf{N}^* > \mathbf{0}$ is a feasible equilibrium point if $f_i\left(\mathbf{N}^*\right) = 0$ for all i.

Around the equilibrium, the trajectories can be described by considering a linearized system. Suppose that the system is resting at the equilibrium $\mathbf{N}^*$, and that a sufficiently small perturbation is applied at time zero, $\mathbf{N}(0) = \mathbf{N}^* + \mathbf{n}(0)$. Then, by Taylor expansion:

$$\frac{d\mathbf{n}(t)}{dt} \approx \mathbf{J}(\mathbf{N}^*)\mathbf{n}(t) \tag{2}$$

where $\mathbf{J}$ is the Jacobian matrix of the system, $J_{ij} = \partial f_i / \partial N_j$. The "community matrix" $\mathbf{M}$ is the Jacobian evaluated at $\mathbf{N}^*$, and therefore

$$\frac{d\mathbf{n}(t)}{dt} \approx \mathbf{M}\,\mathbf{n}(t) \tag{3}$$

which is a system of homogeneous linear differential equations with constant coefficients. This system has solution

$$\mathbf{n}(t) = e^{\mathbf{M}\,t}\mathbf{n}(0) \tag{4}$$

Moreover, if $\mathbf{M}$ is diagonalizable, it can be decomposed as $\mathbf{Q}^{-1}\mathbf{\Lambda}\mathbf{Q}$, where $\mathbf{\Lambda}$ is a diagonal matrix whose diagonal coefficients are the eigenvalues of $\mathbf{M}$, and $\mathbf{Q}$ is a matrix whose columns are the corresponding right eigenvectors. In this case, the solution becomes:

$$\mathbf{n}(t) = \mathbf{Q}^{-1}e^{\mathbf{\Lambda}t}\mathbf{Q}\mathbf{n}(0) \tag{5}$$

meaning that the small perturbation $\mathbf{n}(t)$ will eventually decay to zero if and only if all the eigenvalues of $\mathbf{M}$ have negative real part. Thus, if we order the eigenvalues according to their real part, $\mathrm{Re}\left(\lambda_{\mathbf{M},1}\right) \geq \mathrm{Re}\left(\lambda_{\mathbf{M},2}\right) \geq \ldots \geq \mathrm{Re}\left(\lambda_{\mathbf{M},S}\right)$, stability is exclusively determined by $\mathrm{Re}\left(\lambda_{\mathbf{M},1}\right)$. If $\mathrm{Re}\left(\lambda_{\mathbf{M},1}\right) < 0$, the equilibrium is stable, and if $\mathrm{Re}\left(\lambda_{\mathbf{M},1}\right) > 0$, the equilibrium is unstable.

Circular law. Take a $S \times S$ matrix $\mathbf{M}$ whose coefficients are sampled independently from the distribution X with mean zero and variance one. Then, as $S \to \infty$, the empirical spectral distribution of $\mathbf{M} / \sqrt{S}$ converges to the uniform distribution on the unit disk in

the complex plane[3]. The empirical spectral distribution (ESD) is the distribution obtained assigning a probability of $1/S$ to each of the eigenvalues of $\mathbf{M}$. The circular law therefore implies that, for S large, the "bulk" of the eigenvalues of $\mathbf{M}$ are uniformly spread over the unit disk. Given that the contribution of each eigenvalue to the density becomes negligible as $S \to \infty$, the circular law does not guarantee that all the eigenvalues are eventually contained in the circle. However, with the additional assumption of X having finite fourth moment, one can prove[4] this stronger result: for $S \to \infty$, all eigenvalues of $\mathbf{M} / \sqrt{S}$ are contained in the disk.

The circular law is universal[3]: as long as $\mathrm{E}[X] = 0$ and $\mathrm{Var}[X] = 1$, the ESD of $\mathbf{M} / \sqrt{S}$ converges to the uniform distribution on the unit disk as $S \to \infty$. Moreover, the circular law holds for partially filled (i.e., with few non-zero coefficients) matrices as long as some mild conditions are met[5].

Elliptic law. Take an $S \times S$ matrix $\mathbf{M}$ whose coefficients are sampled as independent pairs (M_{ij}, M_{ji}) from the bivariate distribution Z, with mean $[0, 0]^t$ and covariance matrix

$$\mathbf{\Sigma} = \begin{bmatrix} 1 & \rho \\ \rho & 1 \end{bmatrix} \tag{6}$$

The diagonal elements are sampled from a distribution with mean zero and finite variance. Then, as $S \to \infty$, the ESD of $\mathbf{M} / \sqrt{S}$ converges to the uniform distribution on the ellipse in the complex plane centered at $(0, 0)$, with horizontal semi-axis $1 + \rho$ and vertical semi-axis $1 - \rho$. Also the elliptic law is universal[6,7].

Applications to the stability of ecological systems. One can use the circular and elliptic laws to estimate the rightmost eigenvalue of sufficiently large community matrices.

May[8] was the first to consider a random community matrix $\mathbf{M}$ with i.i.d. entries. The value of each entry M_{ij} in the matrix is determined in the following way: with probability $(1 - C)$, the entry is set to zero, and with probability C its value is sampled from X, with $\mathrm{E}[X] = 0$ and $\mathrm{Var}[X] = \sigma^2$. Then, given that for biological systems the fourth moment is surely bounded, for S large $\mathrm{Re}\,(\lambda_{\mathbf{M},1}) / \sqrt{S\,\mathrm{Var}[M_{ij}]} \approx 1$, and therefore $\mathrm{Re}\,(\lambda_{\mathbf{M},1}) \approx \sqrt{S\,\mathrm{Var}[M_{ij}]} = \sigma\sqrt{SC}$. Because the matrix $\mathbf{M}' = \mathbf{M} - d\,\mathbf{I}$ has eigenvalues as those of $\mathbf{M}$, but shifted by d to the left, then the equilibrium associated with the community matrix $\mathbf{M}'$ is stable whenever d is sufficiently strong to offset the rightmost eigenvalue of $\mathbf{M}$: $\mathrm{Re}\,(\lambda_{\mathbf{M}',S}) = \mathrm{Re}\,(\lambda_{\mathbf{M},1}) - d < 0$, which becomes $\sigma\sqrt{SC} < d$. This result can be further extended to the case of X having non-zero mean, as long as the row sums are approximately constant[1,2]. Ecologically, this is the case when the community matrix is associated with a food web whose structure is random (in the sense of Erdős-Rényi).

The first application of the elliptic law to ecological systems[9] considered that a consumer-resource interaction would appear in the community matrix as a positive coefficient (M_{ij}, effect of resource j on consumer i) paired with a negative coefficient (M_{ji}). This

sign-pairing automatically produces a negative correlation ($\rho < 0$), which is stabilizing, compared to the case $\rho = 0$ studied by May. Similar to the circular law, the elliptic law can be extended to account for non-zero mean, partially connected matrices, and matrices where we subtract d from the diagonal entries[1,2]. Also for these matrices, a key ingredient of the derivation is the assumption of constant row sums, which, in ecological terms, is akin to having food webs with random structure.

Our main goal here is to study the stability of matrices built starting not from Erdős-Rényi random graphs, but rather a more realistic model for food web structure, the cascade model[10].

Role of diagonal coefficients. In most studies of the stability of large ecological systems, the diagonal elements of the matrix $\mathbf{M}$ are assumed to be constant, $-d$, so that the distribution of eigenvalues is the same of that of the corresponding matrix with 0 on the diagonal, shifted of d to the left. Introducing variation among the diagonal elements—which is sensible from an ecological standpoint—could however alter the results. In particular, the diagonal elements impact both the mean and the variance of the eigenvalues:

$$\mathrm{E}[\lambda_i] = \frac{1}{S}\sum_{i=1}^{S} M_{ii} = \mathrm{E}[M_{ii}] \tag{7}$$

$$\mathrm{Var}[\lambda_i] = \mathrm{Var}[M_{ii}] + (S-1)\,\mathrm{E}[M_{ij}M_{ji}] \tag{8}$$

From these equations it is clear that, if the variance does not grow with S, the variance of the eigenvalues is dominated by $(S-1)\,\mathrm{E}[M_{ij}M_{ji}]$, so that a small amount of variation does not alter qualitatively the predictions for large networks[1].

It should however be noted that the variance of the diagonal elements impacts solely the spread along the real axis, with potential consequences for stability. In fact,

$$\mathrm{Var}[\lambda_i] = \mathrm{Var}[M_{ii}] + (S-1)\,\mathrm{E}[M_{ij}M_{ji}] = \mathrm{Var}[\mathrm{Re}(\lambda_i)] - \mathrm{Var}[\mathrm{Im}(\lambda_i)] \tag{9}$$

such that if $\mathrm{Var}[M_{ii}]$ is large compared to $\mathrm{Var}[\mathrm{Re}(\lambda_i)]$, this could have important consequences for stability.

Deriving robust results for random matrices with variable diagonal elements, albeit still an open problem, would make these methods more relevant for biological systems. Studies measuring the variance of the diagonal elements empirically would be especially valuable. For example, a recent article by James *et al.*[11] attempts an estimation of all the coefficients of the community matrix for several small food webs using empirical data.

Supplementary Note 2: Constructing M

In this section we show how matrix **M** is built. **M** is the community matrix of a food web resting at a feasible equilibrium point. The matrix is constructed in two steps. First, we either generate the adjacency matrix **K** (where $K_{ij} = 1$ means that j consumes i) using a model for food web structure, or we take an empirical adjacency matrix. Second, we assign the coefficients of **M** in pairs: we sample from a bivariate distribution $Z = (X, Y)$ a pair of coefficients (M_{ij}, M_{ji}) whenever $K_{ij} = 1$. The diagonal of the matrix **M** is set to zero. In what follows, we first explain how the adjacency matrices are built, and then we show how the distribution Z is parameterized.

Empirical food webs. We took 15 large published food webs, removed any self-loop (cannibalism) and, in the rare cases of double-arrows (i.e., a eats b, and b eats a), we removed one of the two links at random. For each food web, we report the size S, the number of trophic interactions L, and the connectance $C = L/\binom{S}{2}$ (for a justification of this choice of connectance, see the next section). We also report a measure of how close to being acyclic a food web is. If a food web were acyclic, then it would be possible to order all the species from left to right, such that all links point from left to right. If there were any cycles, at least one link would point backwards. For each web, we report the minimum number of unavoidable "feedback links" we observed when trying to find the ordering of the species that minimize it. The procedure is explained in details in below.

1. Ythan Estuary, the food web of the estuary of the Ythan river on the East coast of Scotland[12]. Once removed three cannibalistic loops, the processed food web is acyclic and it comprises $S = 92$ nodes, $L = 414$ feeding interactions, yielding a connectance $C \approx 0.10$.

2. St. Marks, describing the seagrass community of the Goose Creek Bay in the St. Marks National Wildlife Refuge, Florida[13]. After processing, we have $S = 143$, $L = 1763$, $C \approx 0.17$. The food web, once removed three cannibalistic loops, contains no cycles.

3. Grande Cariçaie, the food web of the marsh at Lake Neuchâtel, Switzerland[14]. Once removed two links due to double arrows, we find $S = 163$, $L = 2084$, $C \approx 0.16$. The nodes can be sorted in a hierarchy such that only ≤ 9 links point backwards. We obtained this hierarchy repeatedly applying an algorithm that tries to minimize the number of "feedback links" (see below). Because the problem is not solvable in polynomial time, we write ≤ 9, to stress that better solutions might exist.

4. Serengeti, the plant-mammal food web of the Serengeti grassland ecosystem of Tanzania[15]. Once removed a cannibalistic loop, we have $S = 170$, $L = 585$, and $C \approx 0.04$. The food web contains no cycles.

5. Flensburg Fjord, the food web of a brackish shallow water inlet on the Baltic Sea, located between Germany and Demark[16]. Once removed two cannibalistic loops

and eight links due to double arrows, we find $S = 180$, $L = 1567$, and $C \approx 0.1$. We find ≤ 2 feedback links.

6. Otago Harbour, the food web of an intertidal mudflat ecosystem in New Zealand[17]. We removed six cannibalistic loops and 62 links due to double arrows, obtaining $S = 180$, $L = 1856$, and $C \approx 0.12$. We find ≤ 31 feedback links.

7. Little Rock Lake, the food web of lake in Wisconsin[18]. Removing 17 loops and 42 links, we obtain $S = 181$, $L = 2316$, and $C \approx 0.14$. We find ≤ 11 feedback links.

8. Sylt the food web of the Sylt tidal basin on the North Sea between Germany and Denmark[19]. In this web, once removed the six loops and 34 links participating in double arrows, $S = 230$, $L = 3298$, and $C \approx 0.12$. We find ≤ 16 feedback links.

9. Caribbean Reef, describing the shelf of the American Virgin Islands[20]. Removing 11 loops and nine links, we find $S = 249$, $L = 3293$, $C \approx 0.11$. We find ≤ 3 feedback links.

10. Kongs Fjorden, the food web of the northwest corner of the Svalbard archipelago[21]. We find $S = 270$, $L = 1632$, $C \approx 0.04$. Once removed the 10 loops and the 5 links participating in double arrows, the food web contains no cycles.

11. Carpinteria Salt Marsh, the salt marsh in Carpinteria, California[22]. We find $S = 273$, $L = 3878$, $C \approx 0.1$. We find ≤ 13 feedback links.

12. San Quintin, the food web of Bahia Falsa, in San Quintin, Baja California[22]. We find $S = 290$, $L = 3934$, $C \approx 0.09$. We find ≤ 12 feedback links.

13. Lough Hyne, describing a marine sea lough in south-west Ireland[23]. We remove 13 loops and 13 links, finding $S = 349$, $L = 5088$, $C \approx 0.08$. We find ≤ 6 feedback links.

14. Punta Banda, the food web of the Estero de Punta Banda, in Baja California[22]. We find $S = 356$, $L = 5291$, $C \approx 0.09$. We find ≤ 14 feedback links.

15. Weddell Sea, a marine food web for the high Antarctic eastern Weddell Sea[24]. We remove 51 loops and 394 double arrows, finding $S = 488$, $L = 15435$, $C \approx 0.13$. We find ≤ 229 feedback links.

Cascade model. To build a food web using the cascade[10] model, we order the S species, $1, \ldots, S$. For each species j, we draw a link from any of the preceding species ($K_{ij} = 1, i < j$) with probability C. Note that when $C = 1$, the upper-triangular part of $\mathbf{K}$ is completely filled with ones. This is a definition of connectance that is ideally suited for dealing with pairs of coefficients, as the maximum number of pairs is $\binom{S}{2}$. As such, the best choice of C when building a food web of size S in which the desired number of links is L is $\hat{C} = L/\binom{S}{2}$.

The cascade model produces directed acyclic graphs, so that all the non-zero coefficients of $\mathbf{K}$ are contained in its upper-triangular part.

The number of connections for a food web produced by the cascade model follows the Binomial distribution with parameters C and $\binom{S}{2}$. The expected number of connections is $\mathrm{E}[L] = C\binom{S}{2}$, and variance is $\mathrm{Var}[L] = \binom{S}{2}C(1-C)$. To avoid the rare cases of matrices with many more (fewer) connections than desired, we rejected any network for which the number of connections produced differed from the expectation by more than two standard deviations. We also rejected any disconnected network. For the cascade model, we ended up rejecting few networks (compared to the niche model below), because the Binomial distribution has a very strong central tendency.

Niche model. Building an adjacency matrix using the niche[25] model requires four steps:

1. Each species j is assigned a "niche value", η_j, sampled from a uniform distribution $\mathcal{U}[0,1]$, and the species are sorted in increasing order.
2. A "niche radius" is obtained for each species: $r_j = \eta_j B$, where B is a value sampled from the beta distribution $\mathcal{B}(1,\beta)$.
3. A "niche center", c_j, is sampled from $\mathcal{U}[r_j/2, \min(\eta_j, 1 - r_j/2)]$.
4. Species j consumes all species k whose niche value is included in an interval: $(c_j - r_j/2) \leq \eta_k \leq (c_j + r_j/2)$.

To ensure that we obtain a connectance that is about right[26], we set $\beta = 1/C - 1$. We removed any cannibalistic loop ($K_{jj} = 1$) and, when we found double arrows (where $K_{ij} = K_{ji} = 1$) we removed one of the two links at random. As for the cascade model, we rejected disconnected networks and those with a number of links that differed from the expectation for the cascade model by more than two standard deviations. This criterion is more selective for the niche model than for the cascade model, as the niche model produces networks with a broader range of number of links.

Variants of the cascade model. The number of resources of species j in the cascade model (i.e., its consumer-degree), L_j, depends on the position of j in the hierarchy and on the connectance: $\mathrm{E}[L_j] = C(j-1)$. If we want to change the degree distribution of the consumers to match that of the niche model, it is sufficient to sample L_j from a Binomial distribution with parameters $(j-1)$ and r_j, where $r_j = \eta_j B$, B is sampled from the Beta distribution $\mathcal{B}(1, 1/C - 1)$, and η_j is the jth niche value obtained by sampling uniformly a value for each species, and then sorting them in increasing order.

In the cascade model, the L_j connections of species j are randomly assigned to the preceding species. We could instead choose an interval of species to be consumed by species j, by sampling a random starting point from the uniform discrete distribution $s_j \sim \mathcal{U}\{1, \ldots, j - L_j\}$, and making j consume all the species from s_j to $s_j + L_j - 1$. In this way, we can build a variant of the cascade model that produces interval networks.

Combining the degree distribution and the intervality properties, we can parameterize the three variants of the cascade model described in the main text.

Empirical distributions. For our results, we need to be able to derive a reasonable distribution $Z = (X, Y)$, from which we will then sample independently the pairs (M_{ij}, M_{ji}) whenever $K_{ij} = 1$. The marginal distribution X is that describing the effects of consumers on resources (and thus we expect $\mu_x < 0$), while the marginal Y describes the effects of resources on consumers ($\mu_y > 0$).

To build Z, we rely on a large database of consumer-resource interactions published by Brose *et al.*[27] (we used the amended version of the database published in 2008). The database comprises more than 16,000 documented interactions between consumers and resources in natural food webs, and contains estimates of the average body mass (in grams) for both consumers and resources.

The database is mostly resolved at the level of species, with exceptions including non-living resources (*Detritus*), higher taxonomic levels (e.g., *Crustacea*, *Insecta*), and unresolved organisms (e.g., "Large terrestrial inverts"). To retain only the high-quality data, we matched the taxonomic information for each species with the ITIS database (`itis.org`), and kept only the records for which: a) the average body mass was present for both resource and consumer (≈13,000 records); b) both resource and consumers were matched to a species, genus, or family in the ITIS database (≈8000 records).

For each interaction between consumer i and resource j, we attempted a parameterization of the corresponding coefficients of a community matrix, M_{ij} and M_{ji}. We use body-mass scaling allometries and a Type I functional response, following closely the work of Tang *et al.*[1]:

$$\begin{cases} M_{ij} \approx -a_{ij} N_i^* \\ M_{ji} \approx e_{ij} a_{ij} N_j^* \end{cases} \tag{10}$$

We thus need to parameterize the equilibrium biomasses, N_i^*, the attack rates a_{ij}, and the efficiencies, e_{ij}. The equation above shows clearly that all the coefficients in the same row of the community matrix are all dependent on the corresponding equilibrium N_i^*, a feature that will be lost when we will sample the coefficients independently. Tang *et al.*[1] studied matrices in which these relationships are retained.

We parameterized the equilibrium values $N_i^* \approx 10^{n_0+g+\epsilon_i} m_i^{g+1}$, where $n_0 = -1.16$, ϵ_i is a Normal random variable with mean 0 and standard deviation 0.2, while g, the main parameter controlling the scaling between the body mass and the equilibrium abundance, was varied between −0.45 and −1.05.

The attack rates a_{ij} were modeled as in Tang *et al.*[1]:

$$a_{ij} = 10^{a_0} m_j \frac{\left(\frac{m_i}{m_j}\right)^{0.46}}{1 + \left(\frac{m_i}{m_j}\right)^2} \tag{11}$$

where m_j is the mass of the consumer, m_i that of the resource, and a_0 was set to -3.5 (as the mass is measured in grams). The efficiencies e_{ij} were sampled from the uniform distribution $\mathcal{U}[0.1, 0.25]$.

For each value of g, we produced 100 parameterizations (all slightly different, as the values of ϵ_j and the e_{ij} are randomly drawn), and rescaled the M_{ij} so to obtain a mean negative effect of $\mu_x = -1$ (allowing for a better comparison between the distributions—and without loss of generality, as multiplying a matrix for a positive constant does not alter its stability properties). The values obtained from these simulations are plotted in Figure 1. For $g \approx -0.5$ we have positive means closer in magnitude to the negative mean (-1), and larger variances. The correlation between M_{ij} and M_{ji} is moderately negative for all values of g.

To take a closer look at the distribution of the (M_{ij}, M_{ji}) pairs, in Figures 2, 3, and 4, we plot their empirical distribution along with summary statistics for three parameterizations using $g = -0.55$, $g = -0.75$, and $g = -0.95$, respectively. In all cases, we find that the marginal distributions are very skewed, and contain a few very large values.

We stored the distributions in Figures 2, 3 and 4 and we used them to parameterize our food webs, i.e., when building a matrix $\mathbf{M}$, we sampled independently each pair of interactions from the discrete distribution Z. As a shorthand, we refer to them as Z_{55}, Z_{75} and Z_{95}, respectively. All the figures in the main text have been generated sampling from Z_{75}, while in Section we show the same Figures, but using Z_{55} and Z_{95}.

Bivariate Normal distributions. Given that the circular[3,5] and elliptic[6,7] laws are universal (i.e., they do not depend on the exact details of the distributions used to parameterize the matrices, but rather only on their means and covariance matrices), we are interested in testing whether our results could also be universal. Hence, we repeated the parameterization of the matrices but sampling the entries from bivariate Normal distributions with the same means and covariance matrices found for Z_{55}, Z_{75} and Z_{95}.

One interesting feature of the Normal case is that it is possible to sample (with low probability, given the values of the empirical means and variances) pairs of coefficients with the same sign. We show that this does not alter our results: our conclusions hold for networks where the interactions are not exclusively of consumer-resource type.

Four-corner distributions. To further probe the generality of our results, we parameterize another class of bivariate distributions besides the empirical ones obtained via body-size scaling and the Normal distribution above. However, there are few bivariate distributions that are flexible enough to accommodate any choice of parameters μ_x, μ_y, σ_x, σ_y and ρ_{xy}.

For example, take a bivariate distribution whose marginals are Log-normal (or Uniform). Then, once μ_x, μ_y, σ_x, and σ_y are chosen, not every value of $\rho_{xy} \in [-1, 1]$ can be achieved.

For this reason, we propose here a very simple bivariate distribution that has good sampling qualities, dubbed the "Four-corner distribution". This distribution takes as parameters a vector of means $[\mu_x, \mu_y]^T$ and the corresponding covariance matrix, defined by σ_x, σ_y and ρ_{xy}. The pairs can take one of only four possible values:

$$Z = (X, Y) = \begin{cases} (\mu_x + \sigma_x, \mu_y + \sigma_y) & \text{w.p. } \frac{1+\rho_{xy}}{4} \\ (\mu_x - \sigma_x, \mu_y + \sigma_y) & \text{w.p. } \frac{1-\rho_{xy}}{4} \\ (\mu_x + \sigma_x, \mu_y - \sigma_y) & \text{w.p. } \frac{1-\rho_{xy}}{4} \\ (\mu_x - \sigma_x, \mu_y - \sigma_y) & \text{w.p. } \frac{1+\rho_{xy}}{4} \end{cases} \tag{12}$$

The distribution is therefore straightforward to parameterize and has a shape (and support) that is very different from the Normal and the empirical distributions above. We show that our results hold when the matrices are built using this distribution and the parameters found for Z_{55}, Z_{75} and Z_{95}.

Parameterization of food webs with cycles. If a food web is acyclic, there is at least one way to order the species so that all the non-zero values in the adjacency matrix **K** are contained in the upper-triangular part. Thus, there is at least one way to sort the species in **M** such that all the upper-triangular coefficients are either 0, or have been sampled from X (the first marginal distribution of $Z = (X, Y)$), while the lower-triangular are either 0, or have been sampled from Y.

Having sorted the species in matrix **M** in this way, we can easily define the parameters that will be used in the rest of the derivation. For a large nework with connectance C, the mean of the upper-triangular coefficients of **M** is $\mu_U = C\,\mu_x$, their variance is ${\sigma_U}^2 = C({\sigma_x}^2 + (1-C)\,{\mu_x}^2)$. Similarly, for the lower-triangular coefficients of **M** we have $\mu_L = C\,\mu_y$, and ${\sigma_L}^2 = C({\sigma_y}^2 + (1-C)\,{\mu_y}^2)$. Finally, the covariance $\text{Cov}(M_{ij}, M_{ji}) = \sigma_U\,\sigma_L\,\rho_{UL} = \text{E}[M_{ij}M_{ji}] - \text{E}[M_{ij}]\,\text{E}[M_{ji}] = C(\rho_{xy}\,\sigma_x\,\sigma_y + \mu_x\,\mu_y) - C^2\,\mu_x\,\mu_y$.

However, a problem arises when cycles are present, as there is no ordering of the species such that all the upper-triangular coefficients of **M** are either 0 or sampled from X. In this case, there is no "natural" ordering of the species, and therefore no natural way of defining μ_U, μ_L, σ_U, σ_L, and ρ_{UL}. Each possible ordering of **M** yields different estimate for the parameters of the distribution, so that we need to choose a "rule" for ordering the species in the food web.

Clearly, the eigenvalues of **M** do not depend on the ordering of the species, such that any reordering leaves them completely unaltered. Our approximation, however, is based on the assumption that the upper-triangular elements of **M** are sampled from a different distribution than that of the lower-triangular ones, and thus any of the $S!$ possible ways of ordering **M** would yield a different parameterization—and consequently a different

approximation.

Ideally, given that we sample a coefficient M_{ij} from X whenever $K_{ij} = 1$, the best possible ordering of the species would be that yielding an adjacency matrix $\mathbf{K}$ with the fewest possible non-zero coefficients in the lower-triangular part of the matrix. Unfortunately, this problem—known in the literature as the "minimum feedback-arc set"—is NP-hard[28], and as such an exhaustive search for the best solution is unfeasible.

Fortunately, a few empirical food webs are completely acyclic, and the others (and those constructed using the niche model) have relatively few "feedback links". To choose a reasonable configuration for $\mathbf{K}$, we ran 10 times with different random seeds the program `fas.c` written by David R. MacIver*. For each food web, we took the best solution out of the 10 runs. We did the same for the webs generated by the niche model.

Once found the "best" ordering, and having sorted $\mathbf{K}$ and $\mathbf{M}$ accordingly, we estimated μ_{U}, μ_{L}, σ_{U}, σ_{L}, and ρ_{UL} using the sample means, variances and covariances for the corresponding upper-triangular (lower-triangular) coefficients in $\mathbf{M}$.

*The program is available at `github.com/DRMacIver/Feedback-Arc-Set`

Supplementary Note 3: The eigenvalues of M

To describe the eigenvalues of **M** (see Section), we follow the strategy illustrated in the main text: we decompose the matrix **M** into the sum of two matrices $\mathbf{M} = \mathbf{A} + \mathbf{B}$, and we study the eigenvalues of **A** and **B** separately. We then combine them to approximate the distribution of the eigenvalues of **M**.

The eigenvalues of A. We start by finding all the eigenvalues of the simplest case of **A** in which $A_{ii} = 0$, $A_{ij} = \mu_{\mathrm{L}} = 1$ if $j < i$, and $A_{ij} = \mu_{\mathrm{U}} = -1$ if $j > i$. Because **A** is skew-symmetric and the diagonal elements are set to zero, all eigenvalues have real part zero. For this matrix, the eigenvalues are known, and we follow closely the proof of Bai & Silverstein[4], which we will then extend to our case of interest. The $S \times S$ matrix **A** is

$$A = \begin{bmatrix} 0 & -1 & -1 & \cdots & -1 \\ 1 & 0 & -1 & \cdots & -1 \\ 1 & 1 & 0 & \cdots & -1 \\ \vdots & \vdots & \vdots & \ddots & \vdots \\ 1 & 1 & 1 & \cdots & 0 \end{bmatrix} \tag{13}$$

The characteristic polynomial of **A** is

$$|\lambda\,\mathbf{I} - \mathbf{A}\,| = D_S = \begin{vmatrix} \lambda & 1 & 1 & \cdots & 1 \\ -1 & \lambda & 1 & \cdots & 1 \\ -1 & -1 & \lambda & \cdots & 1 \\ \vdots & \vdots & \vdots & \ddots & \vdots \\ -1 & -1 & -1 & \cdots & \lambda \end{vmatrix} \tag{14}$$

where $|\cdot|$ stands for the determinant of a square matrix. Adding or subtracting a column (row) from another does not change the determinant, and we exploit this fact to rewrite the determinant in a more convenient form. We subtract from each column the subsequent column (this leaves the last column unchanged):

$$D_S = \begin{vmatrix} \lambda - 1 & 0 & 0 & \cdots & 1 \\ -1-\lambda & \lambda - 1 & 0 & \cdots & 1 \\ 0 & -1-\lambda & \lambda - 1 & \cdots & 1 \\ \vdots & \vdots & \vdots & \ddots & \vdots \\ 0 & 0 & 0 & \cdots & \lambda \end{vmatrix} \tag{15}$$

We now take the Laplace expansion along the first column. In fact, $|\lambda\,\mathbf{I} - \mathbf{A}\,| = (\lambda - 1)|(\lambda\,\mathbf{I} - \mathbf{A})_{\{1,1\}}| + (1 + \lambda)|(\lambda\,\mathbf{I} - \mathbf{A})_{\{2,1\}}|$, where $|(\lambda\,\mathbf{I} - \mathbf{A})_{\{i,j\}}|$ is the determinant of the matrix obtained removing the ith row and the jth column from $(\lambda\,\mathbf{I} - \mathbf{A})$, i.e., the corresponding minor. Notice that $|(\lambda\,\mathbf{I} - \mathbf{A})_{\{1,1\}}|$ is simply the determinant of a matrix like $(\lambda\,\mathbf{I} - \mathbf{A})$ but

with size $S-1$, and as such $|(\lambda\,\mathbf{I}-\mathbf{A})_{\{1,1\}}| = D_{S-1}$. The determinant $|(\lambda\,\mathbf{I}-\mathbf{A})_{\{2,1\}}|$ can be evaluated directly, and is equal to $(1+\lambda)^{S-1}$. Combining these facts, we obtain

$$D_S = (\lambda - 1)D_{S-1} + (1+\lambda)^{S-1} \tag{16}$$

which means that we have a recursive formula for the characteristic polynomial D_S. Solving the recursion, we find

$$|\lambda\,\mathbf{I}-\mathbf{A}\,| = \frac{(\lambda+1)^S + (\lambda-1)^S}{2} \tag{17}$$

Setting the polynomial to zero, we can write

$$\frac{(\lambda+1)^S}{(\lambda-1)^S} = -1 \tag{18}$$

This is a complex equation of degree S. The S roots can be written as:

$$\frac{\lambda+1}{\lambda-1} = e^{i\frac{\pi(2k-1)}{S}} \tag{19}$$

where $k = 1, 2, \ldots, S$. Set $\frac{\pi(2k-1)}{S} = \theta_k$. Then,

$$\lambda = \frac{1+e^{i\theta_k}}{-1+e^{i\theta_k}} = \frac{1+\cos\theta_k + i\sin\theta_k}{-1+\cos\theta_k + i\sin\theta_k} \tag{20}$$

we multiply the numerator and the denominator by $-1+\cos\theta_k - i\sin\theta_k$ (the complex conjugate of the denominator)

$$\lambda = -i\frac{\sin\theta_k}{1-\cos\theta_k} = -i\cot\frac{\theta_k}{2} \tag{21}$$

which leads to the solution

$$\lambda = -i\cot\frac{\pi(2k-1)}{2S} \tag{22}$$

We now extend the results above to study the spectrum of a matrix $\mathbf{A}$ having 0 on the diagonal, $\mu_{\mathrm{U}} = -1$ above the diagonal, and $\mu_{\mathrm{L}} = x > 0$ below the diagonal. Having $\mu_{\mathrm{U}} = -1$ is not restrictive, given that any other matrix $\mathbf{A}$ analyzed here (in which $\mu_{\mathrm{U}} < 0$ can take any value) can be reduced to this case by dividing each element of $\mathbf{A}$ by $-\mu_{\mathrm{U}}$.

$$
\mathbf{A} = \begin{bmatrix} 0 & -1 & -1 & \cdots & -1 \\ x & 0 & -1 & \cdots & -1 \\ x & x & 0 & \cdots & -1 \\ \vdots & \vdots & \vdots & \ddots & \vdots \\ x & x & x & \cdots & 0 \end{bmatrix} \tag{23}
$$

Applying the same procedure we used in the case of $x = 1$, we obtain the characteristic polynomial:

$$
|\lambda\, \mathbf{I} - \mathbf{A}\,| = \frac{(\lambda + x)^S + x(\lambda - 1)^S}{1 + x} \tag{24}
$$

Setting the polynomial to zero, we can write

$$
\frac{(\lambda + x)^S}{(\lambda - 1)^S} = -x \tag{25}
$$

and thus

$$
\frac{\lambda + x}{\lambda - 1} = x^{\frac{1}{S}} e^{i\frac{\pi(2k-1)}{S}} \tag{26}
$$

where $k = 1, 2, \ldots, S$. Set $\frac{\pi(2k-1)}{S} = \theta_k$. Then,

$$
\lambda = 1 + \frac{1 + x}{x^{\frac{1}{S}} e^{i\theta_k} - 1} = \left(1 + \frac{(x+1)\left(x^{\frac{1}{S}} \cos\theta_k - 1\right)}{1 + x^{\frac{2}{S}} - 2x^{\frac{1}{S}} \cos\theta_k}\right) + i\left(\frac{(x+1)x^{\frac{1}{S}} \sin\theta_k}{2x^{\frac{1}{S}} \cos\theta_k - 1 - x^{\frac{2}{S}}}\right) \tag{27}
$$

where the first term is the real, and the second the imaginary part of each of the S eigenvalues.

We now show that all eigenvalues of $\mathbf{A}$ lie on curve describing a circle in the complex plane with center

$$
c = \left(\frac{x + x^{\frac{2}{S}}}{x^{\frac{2}{S}} - 1}, 0\right) \tag{28}
$$

and radius

$$
r = \frac{(x+1)x^{\frac{1}{S}}}{1 - x^{\frac{2}{S}}} \tag{29}
$$

To do so, we can shift and rescale the eigenvalues such that according to our conjecture the transformed eigenvalues lie on the curve describing the unit circle

$$\lambda' = \frac{\lambda - \text{Re}(c)}{r} = \frac{e^{i\theta_k} - x^{\frac{1}{S}}}{e^{i\theta_k} x^{\frac{1}{S}} - 1} = \frac{x^{\frac{1}{S}} \cos\theta_k + i \sin\theta_k}{-1 + x^{\frac{1}{S}} \cos\theta_k + i x^{\frac{1}{S}} \sin\theta_k} \tag{30}$$

As above, we multiply both numerator and denominator by $(-1 + x^{\frac{1}{S}} \cos\theta_k - i x^{\frac{1}{S}} \sin\theta_k)$:

$$\lambda' = \left(\frac{2x^{1/S} - (1 + x^{2/S}) \cos\theta_k}{1 + x^{2/S} - 2x^{1/S} \cos\theta_k} \right) + i \left(\frac{(x^{2/S} - 1) \sin\theta_k}{1 + x^{2/S} - 2x^{1/S} \cos\theta_k} \right) \tag{31}$$

Where the first term denotes the real part of λ', while the second term its imaginary part. Computing $\text{Re}(\lambda')^2 + \text{Im}(\lambda')^2$, we obtain:

$$\left(\frac{2x^{1/S} - (1 + x^{2/S}) \cos\theta_k}{1 + x^{2/S} - 2x^{1/S} \cos\theta_k} \right)^2 + \left(\frac{(x^{2/S} - 1) \sin\theta_k}{1 + x^{2/S} - 2x^{1/S} \cos\theta_k} \right)^2 = 1 \tag{32}$$

which proves that all λ' lie on the curve defining the unit circle, and thus the eigenvalues of $\mathbf{A}$ lie on the curve describing the circle with center c and radius r.

The value of x determines the "orientation" of the eigenvalue distribution: if $x < 1$ (Figure 5, left), then we have few eigenvalues with large modulus ($|\lambda| = \sqrt{\text{Re}(\lambda)^2 + \text{Im}(\lambda)^2}$) and negative real part, and many eigenvalues with positive real part. When $x > 1$ (Figure 5, right), we have the reverse.

Thus, for matrices $\mathbf{A}$ in which the negative coefficients are stronger than the positive ones, we expect the stability to be driven by the eigenvalue(s) closest to $\text{Re}(c) + r = 1 - (1 + x)/(x^{1/S} + 1)$. Taking the limit for $S \to \infty$, we find $\text{Re}(\lambda_{\mathbf{A},1}) = (1 - x)/2$, which cannot exceed 1/2. In this case, $\text{Re}(\lambda_{\mathbf{A},1})$ is largest when x is small, which can be interpreted ecologically as extremely low transformation efficiency.

When on the other hand, the positive effects are stronger than the negatives, we find that two roots with large imaginary part are key for stability. From Eq. 27, we find two complex conjugate eigenvalues with the same real part:

$$\lambda_{\mathbf{A},1-2} = \left(1 + \frac{1 + x}{x^{1/S}} \cos\frac{\pi}{S} \right) \pm i \left(\frac{1 + x}{x^{1/S}} \sin\frac{\pi}{S} \right) \tag{33}$$

We conclude this section by summarizing the results for matrix $\mathbf{A}$ in which the upper-triangular elements are all $\mu_{\text{U}} < 0$, the lower-triangular are all $\mu_{\text{L}} > 0$ and $\mu_{\text{U}} \neq -\mu_{\text{L}}$. All the eigenvalues lie on the curve describing a circle in the complex plane centered at

$$c_A = \left(\frac{\mu_{\mathrm{L}} - \mu_{\mathrm{U}} \left(-\frac{\mu_{\mathrm{L}}}{\mu_{\mathrm{U}}}\right)^{2/S}}{\left(-\frac{\mu_{\mathrm{L}}}{\mu_{\mathrm{U}}}\right)^{2/S} - 1}, 0 \right) \tag{34}$$

and with radius

$$r_A = \frac{(\mu_{\mathrm{U}} - \mu_{\mathrm{L}}) \left(-\frac{\mu_{\mathrm{L}}}{\mu_{\mathrm{U}}}\right)^{1/S}}{\left(-\frac{\mu_{\mathrm{L}}}{\mu_{\mathrm{U}}}\right)^{2/S} - 1} \tag{35}$$

(to obtain these values, substitute $x = \mu_{\mathrm{L}} / - \mu_{\mathrm{U}}$ in Eq. 28 and Eq. 29, and multiply by $-\mu_{\mathrm{U}}$). There are two cases: when $-\mu_{\mathrm{U}} > \mu_{\mathrm{L}}$, the circle has center in the left half-plane, most eigenvalues have small modulus and positive real part, while few eigenvalues are large in modulus and have negative real part. When $-\mu_{\mathrm{U}} < \mu_{\mathrm{L}}$, we have the reverse. The case of $-\mu_{\mathrm{U}} = \mu_{\mathrm{L}}$ is equivalent to that studied by Bai & Silverstein[4] and can be seen as a degenerate case in which the circle has infinite radius.

When $-\mu_{\mathrm{U}} > \mu_{\mathrm{L}}$, we can write an inequality for the rightmost eigenvalue:

$$\mathrm{Re}\,(\lambda_{\mathbf{A},1}) \leq \mathrm{Re}(c_A) + r_A = \frac{\mu_{\mathrm{U}} - \mu_{\mathrm{L}}}{\left(-\frac{\mu_{\mathrm{L}}}{\mu_{\mathrm{U}}}\right)^{1/S} + 1} - \mu_{\mathrm{U}} \leq -\mu_{\mathrm{U}} \tag{36}$$

with equality when S is odd. In the limit of large S, we have $\mathrm{Re}\,(\lambda_{\mathbf{A},1}) = (-\mu_{\mathrm{L}} - \mu_{\mathrm{U}})/2$, and therefore the value is bounded by $-\mu_{\mathrm{U}}/2$ from above.

Adding randomness: the "eyeball" matrix. We now want to add randomness to the matrix we constructed above. We can do this in three ways: i) we can make the matrix above partially connected; ii) we can sample the coefficients from distributions, rather than having them all equal to μ_{U} or μ_{L}; iii) we can combine the two types of randomness.

First, we build a random matrix in which the (M_{ij}, M_{ji}) pairs are set to $(-1, x)$ with probability C and are $(0, 0)$ otherwise. In Figure 6 left, we show the eigenvalues of a matrix with $S = 250$, $x = 0.05$ and $C = 0.5$. We can see that the large eigenvalues with negative real part are close to what we would expect from a matrix having $-C$ above the diagonal and xC below the diagonal. The small eigenvalues closer to the real axis, on the other hand, deviate considerably from the prediction, and seem to fall in an ellipse.

We can also add randomness by sampling all the coefficients from distributions. For example, sample those in the upper-triangular part from a Normal distribution with mean μ_{U} and variance σ_{U}^2, and those in the lower-triangular part from a distribution with mean μ_{L} and variance σ_{L}^2. Also in this case (Figure 6, right), we find that the large eigenvalues are well approximated by Eq. 27, while the smaller eigenvalues are well described by an ellipse.

We call these random matrices "eyeball matrices", as their spectrum resembles the structure of the human eye, with the circle describing the sclera and the smaller ellipse corresponding to the iris. When the mean of the upper-triangular elements has larger magnitude than that of the lower-triangular ones ($-\mu_U > \mu_L$), the eyeball is looking towards the right, while when $-\mu_U < \mu_L$ it is looking towards the left.

In these numerical simulations, we observe that, even when randomness is introduced, the eigenvalues with large modulus are still close to the predicted curve for the corresponding **A**, while the smaller ones fall approximately in an ellipse. This is the foundation of our strategy: study the eigenvalues of **A** and **B** separately, and then combine the two distributions to approximate the eigenvalues of **M**.

The eigenvalues of B. The coefficients of the matrix **B** are as those of **M**, but all the upper-triangular ones are obtained by subtracting μ_U, and all the lower-triangular ones by subtracting μ_L. Clearly, this does not change the covariance matrix describing the pairs (B_{ij}, B_{ji}). In fact, we can think of **B** as a matrix built by sampling the pairs of coefficients from a bivariate distribution with means $[0, 0]^t$ and covariance matrix

$$\mathbf{\Sigma} = \begin{bmatrix} {\sigma_U}^2 & \sigma_U \, \sigma_L \, \rho_{UL} \\ \sigma_U \, \sigma_L \, \rho_{UL} & {\sigma_L}^2 \end{bmatrix} \tag{37}$$

If $\sigma_U = \sigma_L = \sigma$, then the solution is quite simple, as, for large S, the eigenvalues of **B** would follow approximately the elliptic law, and thus would be about uniformly distributed in an ellipse in the complex plane, with horizontal semi-axis $r_{B,h} \approx \sqrt{S}\sigma(1 + \rho)$ and vertical semi-axis $r_{B,v} \approx \sqrt{S}\sigma(1-\rho)$. However, typically we have $\sigma_U \neq \sigma_L$, and therefore we need a new conjecture.

We start by making three assumptions:

a) The eigenvalues of **B**, for S large, are approximately uniformly distributed over an ellipse centered at $(0, 0)$, and with semi-axes $r_{B,h}$ and $r_{B,v}$.

b) The sum of the two semi-axes, $r_{B,h} + r_{B,v}$, does not depend on ρ_{UL}, but only on the variances and the size of the matrix.

c) The square of each semi-axis is a second-order polynomial in ρ_{UL} of the form: $r^2_{B,h} = \alpha + \beta \, \rho_{UL} + \gamma \, {\rho_{UL}}^2$, $r^2_{B,v} = \alpha - \beta \, \rho_{UL} + \gamma \, {\rho_{UL}}^2$.

All three assumptions are met in the case of $\sigma_U = \sigma_L = \sigma$ (elliptic law). For this case, we would find that the three assumptions are satisfied, yielding the values $\alpha = \gamma = (S-1)\sigma^2$, and $\beta = 2(S-1)\sigma^2$. In Figures 7, 8 and 9 we show that these are reasonable assumptions even when the condition of equal variances is violated.

Using the three assumptions above, we can solve the values for β and γ for unequal variances ${\sigma_U}^2 \neq {\sigma_L}^2$. First, if the eigenvalues are approximately uniform in an ellipse (assumption a), we can compute the variance of the eigenvalues, $\sum(\lambda_i^2)/S$, as:

$$\int_{-r_{B,h}}^{r_{B,h}} \int_{-r_{B,v}\sqrt{1-\frac{x^2}{r_{B,h}^2}}}^{r_{B,v}\sqrt{1-\frac{x^2}{r_{B,h}^2}}} \frac{1}{\pi\, r_{B,h}\, r_{B,v}} \left(x^2 - y^2\right) dy\, dx = \frac{r_{B,h}^2}{4} - \frac{r_{B,v}^2}{4} \tag{38}$$

where the limits of integration ensure we are integrating over an ellipse, $(\pi\, r_{B,h}\, r_{B,v})^{-1}$ is the density of the uniform distribution over the ellipse, and $(x^2 - y^2)$ descends from the fact that for each $\lambda_1 = x + iy$ we have $\lambda_2 = x - iy$ as well, so that $\lambda_1^2 + \lambda_2^2 = 2x^2 + 2(iy)^2 = 2x^2 - 2y^2$. The variance of the eigenvalues is also equal to

$$\mathrm{Var}(\mathrm{Re}(\lambda)) - \mathrm{Var}(\mathrm{Im}(\lambda)) = \frac{1}{S}\,\mathrm{Tr}(\mathbf{B}^2) = (S-1)\,\sigma_{\mathrm{L}}\,\sigma_{\mathrm{U}}\,\rho_{\mathrm{UL}} \tag{39}$$

which means

$$r_{B,h}^2 - r_{B,v}^2 = 4(S-1)\,\sigma_{\mathrm{U}}\,\sigma_{\mathrm{L}}\,\rho_{\mathrm{UL}} \tag{40}$$

Then, because of assumption c),

$$r_{B,h}^2 - r_{B,v}^2 = 2\beta\,\rho_{\mathrm{UL}} = 4(S-1)\,\sigma_{\mathrm{U}}\,\sigma_{\mathrm{L}}\,\rho_{\mathrm{UL}} \tag{41}$$

and thus $\beta = 2(S-1)\,\sigma_{\mathrm{L}}\,\sigma_{\mathrm{U}}$. If assumption b) is met, then the value of $r_{B,h} + r_{B,v}$ should not depend of ρ_{UL}. We can then equate the value found for $\rho_{\mathrm{UL}} = 0$ with that found for $\rho_{\mathrm{UL}} = 1$. Substituting the value for β, we can write

$$\sqrt{\alpha} + \sqrt{\alpha} = \sqrt{\alpha + 2(S-1)\,\sigma_{\mathrm{U}}\,\sigma_{\mathrm{L}} + \gamma} + \sqrt{\alpha - 2(S-1)\,\sigma_{\mathrm{U}}\,\sigma_{\mathrm{L}} + \gamma} \tag{42}$$

which we can solve for γ:

$$\gamma = \frac{{\sigma_{\mathrm{U}}}^2\,{\sigma_{\mathrm{L}}}^2 (S-1)^2}{\alpha} \tag{43}$$

Substituting γ, we find

$$\begin{cases} r_{B,h} = \frac{\alpha + (S-1)\,\sigma_{\mathrm{L}}\,\sigma_{\mathrm{U}}\,\rho_{\mathrm{UL}}}{\sqrt{\alpha}} \\ r_{B,v} = \frac{\alpha - (S-1)\,\sigma_{\mathrm{L}}\,\sigma_{\mathrm{U}}\,\rho_{\mathrm{UL}}}{\sqrt{\alpha}} \end{cases} \tag{44}$$

To conclude our derivation, we need to find the value of α, which—as we showed above—does not depend on the correlation. As such, we can investigate the case of zero correlation, simplifying the analysis. To derive the value of α, we exploit a recent result

in random matrix theory[29], which allows for the estimation of the largest eigenvalue of a random matrix which is divided into rectangular blocks, and the coefficients are i.i.d. within each block. Using an extension of this approach that takes the limit of infinitely many blocks[30], one can prove that $\alpha \approx \lambda_{\mathbf{G},1}$, where $\lambda_{\mathbf{G},1}$ is the largest eigenvalue of the deterministic matrix

$$\mathbf{G} = \begin{bmatrix} 0 & {\sigma_{\mathrm{U}}}^2 & {\sigma_{\mathrm{U}}}^2 & \cdots & {\sigma_{\mathrm{U}}}^2 \\ {\sigma_{\mathrm{L}}}^2 & 0 & {\sigma_{\mathrm{U}}}^2 & \cdots & {\sigma_{\mathrm{U}}}^2 \\ {\sigma_{\mathrm{L}}}^2 & {\sigma_{\mathrm{L}}}^2 & 0 & \cdots & {\sigma_{\mathrm{U}}}^2 \\ \vdots & \vdots & \vdots & \ddots & \vdots \\ {\sigma_{\mathrm{L}}}^2 & {\sigma_{\mathrm{L}}}^2 & {\sigma_{\mathrm{L}}}^2 & \cdots & 0 \end{bmatrix} \tag{45}$$

Without loss of generality, we can divide by ${\sigma_{\mathrm{U}}}^2$ and define $x = {\sigma_{\mathrm{L}}}^2 / {\sigma_{\mathrm{U}}}^2$, obtaining the matrix

$$\widetilde{\mathbf{G}} = \begin{bmatrix} 0 & 1 & 1 & \cdots & 1 \\ x & 0 & 1 & \cdots & 1 \\ x & x & 0 & \cdots & 1 \\ \vdots & \vdots & \vdots & \ddots & \vdots \\ x & x & x & \cdots & 0 \end{bmatrix} \tag{46}$$

whose spectrum can be studied in exactly the same way we approached the spectrum of $\mathbf{A}$. We can write the characteristic polynomial as a recurrence equation:

$$D_S = (\lambda + 1)D_{S-1} - (\lambda + x)^{S-1} \tag{47}$$

Solving the recurrence, we find:

$$D_S = \frac{x(\lambda + 1)^S - (\lambda + x)^S}{x - 1} \tag{48}$$

Setting the polynomial to zero, we can solve for the largest real root $\lambda_{\widetilde{\mathbf{G}},1}$, which is the Perron eigenvalue of the nonnegative matrix $\widetilde{\mathbf{G}}$:

$$\frac{\lambda_{\widetilde{\mathbf{G}},1} + x}{\lambda_{\widetilde{\mathbf{G}},1} + 1} = x^{1/S} \tag{49}$$

obtaining

$$\lambda_{\widetilde{\mathbf{G}},1} = \frac{x - x^{1/S}}{x^{1/S} - 1} \tag{50}$$

Taking the limit of $\lambda_{\tilde{\mathbf{G}},1}/S$ for $S \to \infty$, we find

$$\lim_{S\to\infty} \frac{\lambda_{\tilde{\mathbf{G}},1}}{S} = \lim_{S\to\infty} \frac{x - x^{1/S}}{S(x^{1/S} - 1)} = \frac{x-1}{\log(x)} \tag{51}$$

so that

$$\alpha \approx S\,\sigma_{\mathrm{U}}^2 \frac{(x-1)}{\log(x)} = S\frac{({\sigma_{\mathrm{U}}}^2 - {\sigma_{\mathrm{L}}}^2)}{\log\left(\frac{{\sigma_{\mathrm{U}}}^2}{{\sigma_{\mathrm{L}}}^2}\right)} = S\frac{({\sigma_{\mathrm{L}}}^2 - {\sigma_{\mathrm{U}}}^2)}{\log\left(\frac{{\sigma_{\mathrm{L}}}^2}{{\sigma_{\mathrm{U}}}^2}\right)} \tag{52}$$

Note that taking the limit for $\sigma_{\mathrm{L}} \to \sigma_{\mathrm{U}}$ we recover $\alpha/S = 1$, consistently with the circular and elliptic law.

To show the quality of the approximation, we plot the predicted ellipse for several cases of bivariate distributions with different marginals in Figures 10 and 11 (Normal distribution), and 12 and 13 (non-Normal marginals).

Combining the distributions. Finally, we need to combine the two eigenvalue distributions. We are inspired by Weyl's inequality: if $\mathbf{M} = \mathbf{A} + \mathbf{B}$, and all matrices were symmetric, then $\mathrm{Re}\,(\lambda_{\mathbf{M},1}) \leq \mathrm{Re}\,(\lambda_{\mathbf{A},1}) + \mathrm{Re}\,(\lambda_{\mathbf{B},1})$. However, this type of inequality has never been applied to the case of non-symmetric matrices of the type studied here.

When we build $\mathbf{M}$ using the cascade model as a starting point, the joint distribution of the pairs $(M_{ij}, M_{ji})_{i<j}$ is given by the bivariate distribution with mean $[\mu_{\mathrm{U}}, \mu_{\mathrm{L}}]^t$ and covariance matrix

$$\mathbf{\Sigma} = \begin{bmatrix} {\sigma_{\mathrm{U}}}^2 & \sigma_{\mathrm{U}}\,\sigma_{\mathrm{L}}\,\rho_{\mathrm{UL}} \\ \sigma_{\mathrm{U}}\,\sigma_{\mathrm{L}}\,\rho_{\mathrm{UL}} & {\sigma_{\mathrm{L}}}^2 \end{bmatrix} \tag{53}$$

where, for S large, $\mu_{\mathrm{U}} = C\,\mu_{\mathrm{x}}$, $\mu_{\mathrm{L}} = C\,\mu_{\mathrm{y}}$, ${\sigma_{\mathrm{U}}}^2 = C({\sigma_{\mathrm{x}}}^2 + (1-C)\,{\mu_{\mathrm{x}}}^2)$, and ${\sigma_{\mathrm{L}}}^2 = C({\sigma_{\mathrm{y}}}^2 + (1 - C)\,{\mu_{\mathrm{y}}}^2)$. C is the connectance, and X and Y are the marginals of Z, the distribution we use to sample the non-zero pairs.

When $C = 1$ (completely filled matrix), the mean and covariance matrix of the pairs of $\mathbf{M}$ are the same as those of Z, simplifying the problem. Take $C = 1$, and $\sigma_{\mathrm{U}} = \sigma_{\mathrm{x}} = \sigma_{\mathrm{L}} = \sigma_{\mathrm{y}} = 0$. Then, $\mathbf{M} = \mathbf{A}$, for which we know all the eigenvalues. What happens when we slightly increase the variances? In Figure 14 we show that, when the variances are very small, the eigenvalues of $\mathbf{M}$ are a distorted version of those of $\mathbf{A}$. However, the small eigenvalues do not fall in an ellipse. Hence, in this regime our approximation scheme does not work, and we end up overestimating $\mathrm{Re}\,(\lambda_{\mathbf{M},1})$. When we increase the variances, our approximation becomes more and more accurate (Figure 14).

Fortunately, ecological networks are only scarcely connected ($C \ll 1$), and this usually translates in higher values of σ_{U} and σ_{L}. Take the case of small variances (e.g., ${\sigma_{\mathrm{x}}}^2 \ll {\mu_{\mathrm{x}}}^2$):

then, for any $1 > C > \sigma_x^2 / \mu_x^2$, the variance of the upper-triangular coefficients σ_U^2 is larger for the partially connected food web ($C < 1$) than for a completely connected web with the same distribution for X. To illustrate this point, in Figure 15 we parameterize matrices with the same distributions used for Figure 14, but with connectance $C = 0.9$. This is sufficient to create the "eyeball" pattern, and as such we can approximate $\mathrm{Re}(\lambda_{\mathbf{M},1})$ as the sum $\mathrm{Re}(\lambda_{\mathbf{A},1}) + \mathrm{Re}(\lambda_{\mathbf{B},1})$, yielding very accurate predictions.

In summary, our approximation is accurate when the variances in the upper-triangular and lower-triangular parts of the matrix **M** are sufficiently large, and this can be due to sufficiently large variances in Z, partially connected networks, or a combination of the two. For all the matrices we parameterized using our empirical distributions or distributions with similar parameters, we found that the approximation is extremely accurate.

Supplementary Note 4: Different parameterizations

In this Section, we repeat the simulations shown in the main text, but we change the distribution $Z = (X, Y)$. First, instead of using Z_{75}, we explore the use of Z_{55} and Z_{95}, i.e., the distributions obtained by changing the scaling relation between the average body-size of a species and its equilibrium density. Then, we parameterize a bivariate Normal distribution and a Four-corner distribution such that they have the same means and covariances found for Z_{55}, Z_{75}, and Z_{95}. In this way, we probe whether the results are likely to be "universal" (i.e., once set the means and covariance matrix, the results are independent of the fine details of the distribution—possibly with extra requirements of bounded moments).

Predicting $\mathbf{Re}\,(\lambda_{\mathbf{M},1})$ In Figures 16 and 17 we show the approximations for Z_{55} and Z_{95}. In Figures 18, 19 and 20 when bivariate Normal distributions with the same means and covariance matrix of Z_{55}, Z_{75} and Z_{95} are used. Finally, in Figures 21, 22 and 23 when we parameterize the equivalent Four-corner distributions. In all cases we find the same qualitative result: the new approximation predicts $\mathrm{Re}\,(\lambda_{\mathbf{M},1})$ of the matrices generated by the cascade model with great accuracy. In all cases, the matrices built using the niche or empirical adjacency matrices are slightly more likely to be stable (on average) than what predicted.

Cascade model variants. To test the validity of the results illustrated in Figure 3 of the main text, we repeated the simulations, but parameterizing the matrices using Z_{55} (Figure 24) and Z_{95} (Figure 25). Also in these cases, we find that adding intervality (and to a lesser extent, a niche-like degree distribution for the consumers) to the cascade model recapitulates the results observed for the niche model. Using a Normal or Four-corner distribution produces the same result.

Empirical food webs and degree distribution. In a similar spirit, we can ask whether altering the degree distribution of the cascade model so that it matches that of an empirical food web can explain the small discrepancy between our prediction and what observed for the matrices parameterized using an empirical adjacency matrix.

To test this hypothesis, we build matrices using a variant of the cascade model in which we provided an empirical degree distribution for the consumers, matching that found for one of the empirical matrices. In Figure 26 we show that these matrices have stability properties very similar to the corresponding empirical ones.

Sensitivity of $\mathbf{Re}\,(\lambda_{\mathbf{M},1})$ **to change in parameters.** In Figure 4 in the main text, we showed that changing the means of interaction strengths had little effect on our approximation of $\mathrm{Re}\,(\lambda_{\mathbf{M},1})$, compared to the effect of changing the variances and the correlation. This pattern is consistent for all parameterizations. One interesting result is that, in partially connected matrices, changing the mean μ_x influences non only the mean of the corresponding part of the matrix, μ_U, but also its variance: $\sigma_U{}^2 \approx C(\sigma_x{}^2 + (1 - C)\,\mu_x{}^2)$. Hence, we would expect the effect of changing means on $\mathrm{Re}\,(\lambda_{\mathbf{M},1})$ to be more muted when the connectance is close

to 1. This point is exemplified in Figure 27, where we show the effect of changing means when the connectance is low (left) or high (right).

Supplementary Note 5: Software

An R package (`eyeball`), which can be used to build the matrices **M** introduced in Section , and to compute all the approximations developed here is available at `https://github.com/StefanoAllesina/eyeball`.

Supplementary References

1. Tang, S., Pawar, S., and Allesina, S. Correlation between interaction strengths drives stability in large ecological networks. *Ecology Letters* **17**(9), 1094–1100 (2014).

2. Allesina, S. and Tang, S. The stability-complexity relationship at age 40. A Random Matrix perspective. *Population Ecology* **57**, 63–75 (2015).

3. Tao, T., Vu, V., and Krishnapur, M. Random matrices: Universality of ESDs and the circular law. *The Annals of Probability* **38**(5), 2023–2065 (2010).

4. Bai, Z. and Silverstein, J. W. *Spectral analysis of large dimensional random matrices*. Springer, (2009).

5. Wood, P. M. Universality and the circular law for sparse random matrices. *The Annals of Applied Probability* **22**(3), 1266–1300 (2012).

6. Naumov, A. Elliptic law for real random matrices. *arXiv* **preprint 1201.1639** (2012).

7. Nguyen, H. and O'Rourke, S. The elliptic law. *Int Math Res Notice* **in press** (2014).

8. May, R. M. Will a large complex system be stable? *Nature* **238**, 413–414 (1972).

9. Allesina, S. and Tang, S. Stability criteria for complex ecosystems. *Nature* **483**(7388), 205–208 (2012).

10. Cohen, J. E., Briand, F., and Newman, C. M. *Community food webs: Data and theory*. Springer-Verlag, Berlin, (1990).

11. James, A., Plank, M. J., Rossberg, A. G., Beecham, J., Emmerson, M., and Pitchford, J. W. Constructing random matrices to represent real ecosystems. *The American Naturalist* ***in press*** (2015).

12. Cohen, J. E., Schittler, D. N., Raffaelli, D. G., and Reuman, D. C. Food webs are more than the sum of their tritrophic parts. *Proceedings of the National Academy of Sciences* **106**(52), 22335–22340 (2009).

13. Christian, R. R. and Luczkovich, J. J. Organizing and understanding a winter's seagrass foodweb network through effective trophic levels. *Ecological Modelling* **117**(1), 99–124 (1999).

14. Cattin Blandenier, M.-F. *Food web ecology: models and application to conservation*. PhD thesis, (2004).

15. Baskerville, E. B., Dobson, A. P., Bedford, T., Allesina, S., Anderson, T. M., and Pascual, M. Spatial guilds in the Serengeti food web revealed by a Bayesian group model. *PLoS computational biology* **7**(12), e1002321 (2011).

16. Zander, C. D., Josten, N., Detloff, K. C., Poulin, R., McLaughlin, J. P., and Thieltges, D. W. Food web including metazoan parasites for a brackish shallow water ecosystem in Germany and Denmark: Ecological Archives E092-174. *Ecology* **92**(10), 2007–2007 (2011).

17. Mouritsen, K. N., Poulin, R., McLaughlin, J. P., and Thieltges, D. W. Food web including metazoan parasites for an intertidal ecosystem in New Zealand: Ecological Archives E092-173. *Ecology* **92**(10), 2006–2006 (2011).

18. Martinez, N. D. Artifacts or attributes? Effects of resolution on the Little Rock Lake food web. *Ecological Monographs* **61**, 367–392 (1991).

19. Thieltges, D. W., Reise, K., Mouritsen, K. N., McLaughlin, J. P., and Poulin, R. Food web including metazoan parasites for a tidal basin in Germany and Denmark: Ecological Archives E092-172. *Ecology* **92**(10), 2005–2005 (2011).

20. Opitz, S. *Trophic interactions in Caribbean coral reefs*. Number 1085. WorldFish, (1996).

21. Jacob, U., Thierry, A., Brose, U., Arntz, W. E., Berg, S., Brey, T., Fetzer, I., Jonsson, T., Mintenbeck, K., Mollmann, C., et al. The role of body size in complex food webs: A cold case. *Advances In Ecological Research* **45**, 181–223 (2011).

22. Hechinger, R. F., Lafferty, K. D., McLaughlin, J. P., Fredensborg, B. L., Huspeni, T. C., Lorda, J., Sandhu, P. K., Shaw, J. C., Torchin, M. E., Whitney, K. L., et al. Food webs including parasites, biomass, body sizes, and life stages for three California/Baja California estuaries: Ecological Archives E092-066. *Ecology* **92**(3), 791–791 (2011).

23. Riede, J. O., Brose, U., Ebenman, B., Jacob, U., Thompson, R., Townsend, C. R., and Jonsson, T. Stepping in Elton's footprints: a general scaling model for body masses and trophic levels across ecosystems. *Ecology Letters* **14**(2), 169–178 (2011).

24. Jacob, U. *Trophic dynamics of Antarctic shelf ecosystems: food webs and energy flow budgets*. PhD thesis, Bremen, Univ., Diss., 2005, (2005).

25. Williams, R. J. and Martinez, N. D. Simple rules yield complex food webs. *Nature* **404**(6774), 180–183 (2000).

26. Allesina, S., Alonso, D., and Pascual, M. A general model for food web structure. *Science* **320**(5876), 658–661 (2008).

27. Brose, U., Cushing, L., Berlow, E. L., Jonsson, T., Banasek-Richter, C., Bersier, L.-F., Blanchard, J. L., Brey, T., Carpenter, S. R., Blandenier, M.-F. C., et al. Body sizes of consumers and their resources: Ecological Archives E086-135. *Ecology* **86**(9), 2545–2545 (2005).

28. Karp, R. M. *Reducibility among combinatorial problems*. Springer, (1972).

29. Aljadeff, J., Renfrew, D., and Stern, M. Eigenvalues of block structured asymmetric random matrices. *arXiv* **preprint 1411.2688** (2014).

30. Aljadeff, J., Renfrew, D., and Sharpee, T. Why do receptive field models work in recurrent networks? *Cosyne Abstracts, Salt Lake City USA* (2015).
